# Dietary patterns and the progression from obesity to sarcopenic obesity in older adults: a prospective cohort study

**DOI:** 10.1186/s12877-026-07857-1

**Published:** 2026-06-25

**Authors:** Shu-Yi Li, Jason Leung, Ting Zhang, Yafei Wu, Blanche Yu, Timothy Kwok

**Affiliations:** 1 Department of Medicine and Therapeutics, Faculty of Medicine, The Chinese University of Hong Kong, Shatin, Hong Kong China; 2 Jockey Club Centre for Osteoporosis Care and Control, The Chinese University of Hong Kong, Hong Kong, China; 3 Department of Geriatrics, Renji Hospital, School of Medicine, Shanghai Jiaotong University, Shanghai, China; 4 Faculty and Planning Office, Faculty of Medicine, The Chinese University of Hong Kong, Hong Kong, China

**Keywords:** Dietary pattern, Sarcopenic obesity, Inflammation, Older adults, Prospective cohort

## Abstract

**Background:**

Evidence on dietary patterns and incident sarcopenic obesity (SO) remains limited, and whether associations vary by baseline body composition status is unclear. This study aimed to investigate associations between dietary patterns and incident SO and examine potential effect modification by baseline status (normal, obesity only, or sarcopenia only).

**Methods:**

This study included 3,398 community-dwelling Chinese older adults free of SO at baseline. Dietary data was assessed using a 280-item validated quantitative food frequency questionnaire (FFQ); a priori dietary indices (Diet Quality Index-International [DQI-I], Mediterranean-DASH Intervention for Neurodegenerative Delay [MIND], Okinawa diet score, and Dietary Inflammatory Index [DII]) were calculated, and *a posteriori* dietary patterns were derived using factor analysis. SO was defined as the coexistence of sarcopenia (Asian Working Group for Sarcopenia 2025 criteria) and obesity (based on body fat percentage). Cox proportional hazards models were used to estimate hazard ratios (HRs) for incident SO.

**Results:**

During four years of follow-up, 266 participants developed SO, with approximately 80% of incident cases occurring among participants with baseline obesity. Higher DQI-I (HR 0.854, 95% CI 0.756–0.964 per SD increase), MIND (HR 0.853, 95% CI 0.754–0.965), and Okinawa (HR 0.866, 95% CI 0.764–0.982) scores were associated with a lower risk of incident SO, whereas higher DII scores increased incident SO risk (HR 1.158, 95% CI 1.007–1.332). In stratified analyses, associations for antioxidant-rich (higher MIND and Okinawa scores) and pro-inflammatory (higher DII) diets were significant only among participants with baseline obesity, with no significant associations in those with normal body composition or sarcopenia only.

**Conclusions:**

Adherence to dietary patterns with low inflammatory potential and high antioxidant capacity may mitigate the progression from obesity to SO in older adults.

**Supplementary Information:**

The online version contains supplementary material available at https://doi.org/10.1186/s12877-026-07857-1.

## Introduction

Population aging is accelerating globally and is accompanied by a rising burden of age-related diseases. Among these, sarcopenic obesity (SO), defined as the co-occurrence of sarcopenia and obesity, has emerged as a critical geriatric syndrome, affecting approximately 10% of older adults [1–3]. Individuals with SO face significantly higher risks of cardiovascular disease (CVD), metabolic syndrome, and physical disability, compared with individuals who have either sarcopenia or obesity alone [4, 5]. Optimal prevention and management strategies for SO differ from those for sarcopenia or obesity alone [6, 7]. While sarcopenia prevention primarily focuses on stimulating muscle synthesis, SO poses the additional challenge of reducing fat mass while preserving muscle mass and function. Given that pharmacological interventions remain in their early stage, current approaches rely on lifestyle modifications [2, 7]. Diet is a key modifiable factor and offers a promising strategy for the prevention and management of SO [8].

Previous nutritional epidemiology studies have focused on single nutrients, such as protein, leucine, or vitamin D [9–12]. However, this may overlook synergistic interactions among foods and nutrients within the overall diet. More attention has shifted toward dietary patterns, which better reflect real-world consumption [13–18]. Dietary patterns can be defined by two primary approaches: *a priori *and *a posteriori* methods [19]. *A priori* methods quantify dietary adherence using predefined scoring systems based on established dietary guidelines [19], such as the Mediterranean diet score (MDS) [20], and the Dietary Approaches to Stop Hypertension (DASH) diet [21]. In contrast, *a posteriori* methods are data-driven and capture the specific habitual dietary behaviors of a given regional population [19]. By utilizing both approaches, the impact of both recognized dietary recommendations and region-specific dietary habits on the risk of incident SO can be comprehensively evaluated. Although several studies suggest these healthy patterns are inversely associated with SO risk, others have reported inconsistent or null associations [15, 18]. In addition, most previous studies are cross-sectional designs, which limit the ability to clarify causal inferences [13–18].

A key research gap remains in clarifying the developmental trajectories leading to SO and the role of diet in development of SO. SO may come from: (1) the “obesity-to-SO” pathway, in which adipose-derived inflammation and lipotoxicity impair mitochondrial function, accelerating muscle atrophy [3]; and (2) the “sarcopenia-to-SO” pathway, in which muscle loss reduces energy expenditure and physical activity, leading to fat accumulation [22]. These distinct pathways suggest that dietary strategies may need to be tailored to baseline body composition status. Individuals with baseline obesity may benefit more from anti-inflammatory dietary patterns to mitigate obesity-related inflammation, whereas those with baseline sarcopenia may benefit more from adequate protein intake and an overall balanced dietary pattern to support muscle protein synthesis and limit fat gain. However, most studies examining dietary patterns in relation to incident SO have not stratified participants by baseline body composition status (sarcopenia only, obesity only, or normal). As a result, observed associations may be diluted, and potential subgroup-specific protective effects of dietary patterns may be obscured.

Therefore, the primary aim of this study was to investigate the prospective association between dietary patterns and incident SO. As a secondary aim, we conducted subgroup analyses stratified by baseline status (sarcopenia only, obesity only, or normal) to examine whether associations differ according to baseline body composition status and subsequent progression to SO.

## Methods

### Study participants

This was a prospective cohort study. Data in this study were from the Mr. OS and Ms. OS (Hong Kong) cohort [23, 24]. A total of 4,000 Chinese men and women aged 65 years or above were recruited between 2001 and 2003 by health talks or advertisement in the local communities. Participants were followed up at the 2-year (2003–2005) and the 4-year (2005–2007) follow-up visit. All data were collected at the research center, where trained research staff conducted face-to-face interviews and clinical examinations. For the present analysis, individuals were excluded if they lacked dietary intake information (*n* = 5), reported implausible energy intake (> 3,500 kcal/day for women and > 4,000 kcal/day for men, or < 500 kcal/day for women and < 800 kcal/day for men) (*n* = 13) [25], had SO at baseline (*n* = 174), or had no follow-up data (*n* = 410). A final sample of 3,398 participants remained for the prospective association of dietary patterns and incident SO. The flowchart of study participants was shown in Supplementary Figure S1. Ethical approval for this study was obtained from the Clinical Research Ethics Committee of The Chinese University of Hong Kong. All participants were informed of the study details and provided written consent before participation.

### Dietary data collection

Baseline dietary intake was assessed using a 280-item quantitative food frequency questionnaire (FFQ) administered through face-to-face interviews by trained research staff. This FFQ, previously validated in a local population [26], was designed to capture habitual dietary intake over the past 12 months. It includes the following categories of foods: bread/pasta/rice, vegetables, fruits, meat/fish/eggs, beverages, dim sum/snacks, soup, and oil/salt/sauces. Participants reported intake frequency and portion sizes for each item, helped by a visual guidebook featuring standardized food portion photographs to enhance reporting accuracy. Nutrient and energy intakes were derived using the Chinese Food Composition Table and the McCance and Widdowson’s composition of foods [27, 28]. Dietary intakes were energy-adjusted using the residual method to account for variations in energy intake [29].

### Dietary patterns

#### A priori dietary pattern score

Based on the nutrient and food intakes estimated by FFQ, six established dietary pattern scores were used in this study, including the Diet Quality Index-International (DQI-I), Dietary Inflammatory Index (DII), MDS, DASH, Mediterranean-DASH Intervention for Neurodegenerative Delay (MIND) diet score, and the Okinawan diet score [42]. The beneficial, moderate, and detrimental dietary components of these dietary patterns are shown in Supplementary Table S1. The DQI-I assesses overall dietary quality through four components: variety, adequacy, moderation, and balance, with a total score ranging from 0 to 100, where higher scores reflect better diet quality [43, 44]. In this study, due to the absence of data on empty-calorie foods, the moderate component was adjusted from 30 to 24, reducing the maximum DQI-I score to 94. The DII was used to estimate the inflammatory potential of a diet, based on a literature-derived scoring algorithm involving 45 dietary parameters and their associations with inflammatory biomarkers [45]. Due to data availability, 30 dietary parameters were included in the calculation of DII [46]. Lower and negative DII scores indicate more anti-inflammatory dietary profiles, and higher and positive scores reflect pro-inflammatory potential. Adherence to the Mediterranean diet was assessed using MDS, calculated according to the revised method by Trichopoulou et al. [20]. Vegetables, legumes/seeds/nuts, fruits, cereals, fish, and the monounsaturated-to-saturated lipid ratio, were given a score of 1 if daily consumption was at or above the sex-specific median. Meat, poultry, and dairy products, received a score of 1 if consumption was below the sex-specific median. The beverage category from FFQ included alcoholic beverages (wine, spirits, light beer, beer, and Chinese wine), which were converted into daily ethanol intake (g/day). In calculating the MDS, a score of 1 was assigned for the ethanol component if daily consumption was within 5–25 g for women and 10–50 g for men. The total MDS ranges from 0 to 9, with higher scores indicating greater adherence. The DASH score was calculated based on the method by Mellen et al. [21], evaluating intake of nine key nutrients: total fat, saturated fat, protein, fiber, cholesterol, calcium, magnesium, potassium, and sodium. The total DASH score ranged from 0 to 9, with higher scores indicating better adherence. The MIND diet incorporates elements from both the Mediterranean and DASH diets, emphasizing neuroprotective foods [47]. It recommends the intake of ten beneficial food categories, including leafy greens, other vegetables, nuts, berries, legumes, whole grains, seafood, poultry, olive oil, and wine, while limiting the consumption of five types of foods such as red meat, butter and margarine, cheese, desserts, and fried fast food. The total MIND score ranged from 0 to 15, with higher scores indicating greater adherence. The Okinawan diet score was based on traditional dietary patterns characterized by low caloric intake and high nutrient density, as described by Willcox et al. [48]. It has been considered beneficial for longevity and includes food group-specific energy contribution targets: sweet potatoes (69%), rice (12%), other grains including wheat and barley (7%), legumes (6%), vegetables (3%), oils (2%), fish (1%), and < 1% each for nuts and seeds, meat, eggs, dairy, fruit, other potatoes, sugars, pickled vegetables, flavors, and alcohol. One point is assigned for each component meeting the traditional Okinawan proportion, with seaweed excluding due to limited data, resulting in a maximum score of 16 in this study. The traditional Okinawan diet relies heavily on sweet potatoes, which differs from the rice-based diet of Hong Kong Chinese. In our cohort, no participants scored ≥ 1 on the sweet potato component. Nevertheless, the Okinawa score was used because it effectively captures other key dietary components and relevant to this study population, such as higher intakes of legumes and vegetables, and lower intake of meat and dairy products.

#### A posterior dietary pattern score

Individual FFQ food items were grouped into 32 categories based on similarity in type and nutrient composition. Each food group was energy adjusted by dividing the energy intake from each food group by total energy intake and multiplying by 100 and was expressed as a percentage of total energy intake. Details of the methodology in dietary pattern scores derived by factor analysis have been published previously [30, 31]. Briefly, factor analysis with varimax rotation was conducted on these 32 food groups. Three factors were retained based on eigenvalues > 1.0, visual inspection of the scree plot, and biological interpretability. Food groups with absolute factor loadings ≥ 0.2 were considered to characterize a pattern. Factor scores were calculated for each subject by summing intakes weighted by their factor loadings. Higher scores indicated greater adherence to the identified pattern. As detailed in Supplementary Table S2, the three identified patterns were: (1) the ‘vegetables-fruits’ pattern, characterized by vegetables, fruits, legumes, soy and soy products; (2) the ‘snacks-drinks-dairy’ pattern, characterized by condiments, coffee, fast food, French fries, potato chips, nuts, dairy products, whole grains, sweets and desserts; and (3) the ‘meat-fish’ pattern, characterized by red and processed meats, poultry, fish and seafood, and dim sum.

### Measurements of body composition and physical performance

Body composition and physical performance were measured at baseline, 2-year and 4-year follow-up visit. Body weight was measured using a calibrated Physician Beam Balance Scale (Healthometer, McCook, IL, USA), and height was assessed using a stadiometer (Holtain Ltd., Crosswell, UK). Body mass index (BMI) was calculated as weight in kilograms divided by the square of height in meters (kg/m^2^). Waist circumference (WC) was measured using a tape measure while participants maintained an upright standing posture. Body composition was assessed using dual-energy X-ray absorptiometry (DXA) scan with a Hologic QDR 4500 W scanner (Hologic Inc., Waltham, MA, USA). Whole-body, trunk, and limb measurements were used to determine lean mass, fat mass, and bone mass. The coefficients of variation were 1.47% for measurements of fat mass and 0.84% for lean mass. Muscle mass was defined as lean mass minus bone mass. Appendicular skeletal muscle mass (ASM) was calculated as the sum of muscle mass in both upper and lower limbs. ASM index (ASMI) is defined as ASM divided by the square of height (kg/m^2^). Body fat percentage (BFP) was calculated as total body fat mass divided by total body weight and multiplied by 100.

Physical performance was evaluated using three indicators: handgrip strength, gait speed, and five-time chair stand test. Grip strength was measured with a JAMAR Hand Dynamometer (Model 5030J1; Sammons Preston, Bolingbrook, IL, USA). Two measurements were taken for each hand, and the highest value was used for analysis. Gait speed was assessed by timing participants as they walked a straight 6-meter path at their usual pace. The five-time chair stand test recorded the time taken to rise from a seated position five consecutive times.

### Definition of sarcopenic obesity

SO was defined according to the consensus statement by the European Society for Clinical Nutrition and Metabolism (ESPEN) and the European Association for the Study of Obesity (EASO), as the co-existence of sarcopenia, characterized by low muscle mass and low muscle strength, and obesity, defined by excessive BFP [2]. Sarcopenia was defined based on the 2025 Asian Working Group for Sarcopenia (AWGS) criteria, which include low muscle mass (ASMI < 7.0 kg/m^2^ for men and < 5.4 kg/m^2^ for women, or ASM/BMI < 0.83 for men and < 0.57 for women) and low grip strength (< 28 kg for men and < 18 kg for women) [32]. Obesity was defined by BFP thresholds recommended by the American Association of Clinical Endocrinology and the American College of Endocrinology, and WHO criteria: BFP ≥ 25% for men and ≥ 35% for women [33]. Participants were classified into four groups based on their sarcopenia and obesity status: normal, sarcopenia alone, obesity alone, and SO.

### Covariates assessment

Sociodemographic characteristics (age, sex, and educational level), lifestyle factors (smoking status and alcohol consumption), and medical history were collected using a structured questionnaire that has been previously described and utilized elsewhere [23, 24]. Physical activity was assessed using the 12-item Physical Activity Scale for the Elderly (PASE) [34], which calculates a weighted summary score based on the frequency and energy expenditure of leisure, household, and occupational activities over the past seven days. Higher PASE scores indicate greater levels of physical activity. Cognitive function was evaluated using the cognitive subscale of the Chinese version of the Community Screening Instrument for Dementia (CSI-D) [35, 36]. The CSI-D is composed of 32 items distributed across six cognitive domains: time orientation, spatial orientation, praxis, abstract reasoning, language, and memory. Probable dementia was defined by a CSI-D score of ≤ 28.4 points. Serum high-sensitivity C-reactive protein (hs-CRP) was measured using the Vitros Fusion 5.1 enzyme-linked immunosorbent assay (Vitros Chemistry Products, Rochester, NY), with analyses conducted by PathLab Co. Ltd. (Council Bluffs, IA). Serum creatinine was quantified using liquid chromatography-tandem mass spectrometry. The estimated glomerular filtration rate (eGFR) was calculated using the Chronic Kidney Disease Epidemiology Collaboration (CKD-EPI) creatinine equation, incorporating serum creatinine, age, and sex [37].

### Statistical analyses

Baseline characteristics of participants were summarized according to incident SO and Non-SO. Continuous variables were presented as means with standard deviations (SD) or medians with interquartile ranges (IQR), while categorical variables were reported as number of participants and percentages. Baseline characteristics were also compared across the three baseline body composition categories (normal, sarcopenia only, and obesity only). Group differences were assessed using *t*-test or the Mann–Whitney *U* test, and analysis of variance (ANOVA) or Kruskal-Wallis tests for continuous variables, and chi-square test for categorical variables.

To estimate the association between dietary pattern scores and incident SO, Cox proportional hazards models were used. Hazard ratios (HRs) and corresponding 95% confidence intervals (95% CIs) were presented. Person-years were calculated for each participant as the date of SO identified or the date of follow-up visit minus the date of baseline. Each dietary pattern score was presented as per SD increase as a continuous variable. Incidence rates of SO were calculated as the number of incident cases divided by the total person-years of follow-up and expressed per 1,000 person-years. Model 1 was the age- and sex-adjusted model. Model 2 was adjusted for age, sex, educational level, living alone, smoking status, alcohol consumption, total energy intake, physical activity level, medical history of hypertension, diabetes and heart diseases, and probable dementia. To account for multiple testing across the nine dietary exposures, we used the Benjamini-Hochberg False Discovery Rate (FDR) procedure to the primary models. Restricted cubic spline (RCS) Cox regression models were performed to plot the potential non-linear associations of dietary pattern scores and incident SO. There were three points located at the 10th, 50th, and 90th percentiles of dietary patten scores, and the median was used as reference in the RCS models with multivariable adjustment. Subgroup analysis based on the baseline sarcopenia and obesity status (normal, sarcopenia only or obesity only) was conducted to examine the association between dietary patterns and incident SO across different trajectories of SO development. To evaluate whether the associations between dietary patterns and incident SO differed by baseline body composition status, interaction tests were conducted by including cross-product terms (dietary pattern score × baseline status) in the models. Furthermore, to explore potential sex differences, stratified analyses by sex were conducted. The potential effect modification by adding an interaction term were tested into the multivariable models.

To address potential exposure misclassification due to dietary changes over time, a sensitivity analysis restricting the follow-up period to 2 years was conducted. To address potential selection bias and informative censoring due to loss to follow-up, an Inverse Probability of Censoring Weighting (IPCW) sensitivity analysis was conducted. We used logistic regression to estimate each participant’s probability of remaining in the study. The primary models were then reanalyzed using the inverse of these probabilities as weights. Furthermore, to quantify the robustness of our significant findings to potential unmeasured confounding or unmeasured selection bias, we calculated E-values for the point estimates. In addition, a sensitivity analysis using the Fine-Gray subdistribution hazard model was conducted to estimate subdistribution hazard ratios (SHRs) and account for the competing risk of death. To test the robustness of our findings against different definitions of obesity and sarcopenia, we conducted additional sensitivity analyses. First, obesity was also defined using the 60th percentile of BFP in our study population [38, 39], corresponding to BFP $\:\ge\:$25.9% for men and $\:\ge\:$36.2% for women. Second, we applied an anthropometry-based definition using the WHO criteria for Asian populations: BMI ≥27.5 kg/m^2^, or WC ≥90 cm (men), ≥80 cm (women) [40]. Third, we compared incident SO cases identified by the AWGS 2025 criteria with those identified by the AWGS 2019 criteria [41]. The main regression analyses using AWGS 2019-defined incident SO as the outcome were repeated.

To reduce residual confounding of inflammation and renal function, sensitivity analyses were also conducted by additionally adjusted for serum hs-CRP or eGFR on the multivariable-adjusted Cox model. In addition, to investigate the interaction between dietary patten scores with serum hs-CRP or eGFR on incident SO, we added multiplicative terms into the multivariable adjusted Cox model. Stratified analyses were performed in subgroups defined by the median of serum hs-CRP or eGFR. In addition, associations between per SD increase in dietary patten scores with log-transformed serum hs-CRP or eGFR were examined by multivariable adjusted linear regression model.

All statistical analyses were conducted using STATA version 17 (StataCorp LLC, College Station, TX, USA) and RStudio version 2024.12.0 + 467 (Posit Software, PBC, Boston, MA, USA). A two-sided *p*-value < 0.05 was considered statistically significant.

## Results

### Baseline characteristics of study participants

The study included 3,398 participants with a mean age of 72.1 (SD 4.9) years and a mean BMI of 23.7 (SD 3.2) kg/m^2^, and 50.4% of study participants were women. Table 1 shows the baseline characteristics stratified by incident SO status (developed SO during follow-up vs. remained non-SO). Participants who developed SO were older; had higher prevalences of CVD and diabetes; reported lower physical activity; had slightly lower CSI-D scores; and showed higher serum hs-CRP levels (all *p* < 0.05), compared with the non-SO group. No significant differences were observed regarding education level, smoking status, alcohol consumption, or eGFR. In addition, those who developed SO had higher WC and BFP, lower ASMI and ASM/BMI, and poorer physical performance (all *p* < 0.001). They also tended to have lower diet quality, MIND and Okinawa diet scores and higher DII scores, compared with the non-SO group. Furthermore, baseline characteristics stratified by body composition status (normal, sarcopenia only, and obesity only) are detailed in Supplementary Table 3. The sarcopenia only group was older with more men and smokers, whereas the obesity only group had higher prevalences of CVD and hypertension, and elevated CRP. Compared to the normal group, both groups had lower physical activity and poorer overall diet quality. Compared with participants included, participants without follow-up information were generally older, less educated, more likely to live alone, and had lower physical activity and energy intake (Supplementary Table S4). They also had lower diet quality (lower DQI, DASH, MIND, and Okinawa diet scores; higher DII scores), lower muscle mass and muscle strength, elevated serum hs-CRP levels, and a higher prevalence of CVD and cognitive impairment.

**Table 1:** Baseline characteristics of study participants

	Non-SO	Incident SO	*p*
*N*	3,132	266	
Age, years	71.88 (4.89)	74.08 (4.95)	< 0.001
Women, *n* (%)	1,577 (50.35)	135 (50.75)	0.900
Education, *n* (%)			0.092
No education	614 (20.47)	57 (21.43)	
Primary or below	1,565 (49.97)	147 (55.26)	
Secondary or above	926 (29.57)	62 (23.31)	
Living alone, *n* (%)	327 (10.44)	29 (10.90)	0.813
Smoking status, *n* (%)			0.518
Non-smokers	2,009 (64.14)	165 (62.03)	
Former smokers	916 (29.25)	88 (32.33)	
Current smokers	207 (6.61)	15 (5.64)	
Alcohol drinking, *n* (%)	431 (13.77)	29 (10.90)	0.190
CSI-D	30.32 (1.92)	30.05 (2.13)	0.028
Probable dementia, *n* (%)	427 (13.63)	43 (16.17)	0.251
CVD, *n* (%)	614 (19.60)	66 (24.81)	0.042
Diabetes mellitus,* n* (%)	437 (13.95)	50 (18.80)	0.030
Hypertension, *n* (%)	1,305 (41.67)	124 (46.62)	0.116
PASE score	94.09 (43.35)	80.42 (38.21)	< 0.001
Weight, kg	58.70 (9.79)	58.12 (8.45)	0.347
BMI, kg/m^2^	23.62 (3.22)	24.42 (3.20)	< 0.001
WC, cm	86.12 (9.37)	88.33 (8.38)	< 0.001
BFP, %	29.01 (7.12)	33.30 (6.75)	< 0.001
ASMI, kg/m^2^	6.69 (0.96)	6.33 (0.93)	< 0.001
ASM/BMI	0.71 (0.14)	0.63 (0.14)	< 0.001
Grip strength, kg	28.97 (8.10)	25.02 (5.94)	< 0.001
Gait speed, m/s	1.03 (0.22)	0.96 (0.21)	< 0.001
Chair stand test, s	12.75 (4.19)	14.16 (5.40)	< 0.001
Sarcopenia, *n* (%)	99 (3.16)	12 (4.51)	0.234
Obesity, *n* (%)	1,385 (44.22)	214 (80.45)	< 0.001
Serum hs-CRP, mg/L	1.60 (0.80–3.30)	1.90 (1.10–3.50)	0.009
eGFR, mL/min/1.73 m^2^	85.06 (73.36–91.08)	83.68 (68.16–91.31)	0.162
Energy intake, kcal/day	1852.66 (574.26)	1801.39 (589.81)	0.163
Dietary pattern scores			
DQI-I	64.9 (9.35)	62.95 (10.30)	0.001
DII	−0.55 (1.45)	−0.26 (1.60)	0.002
MDS	4.11 (1.53)	3.97 (1.54)	0.151
DASH	4.00 (1.27)	3.99 (1.29)	0.910
MIND diet	4.63 (0.93)	4.43 (0.92)	0.001
Okinawa diet	7.22 (1.39)	6.97 (1.36)	0.006
*Hong Kong diet pattern*			
Factor 1: vegetables-fruits	0.015 (0.993)	−0.034 (1.027)	0.439
Factor 2: snacks-drinks-milk products	0.023 (1.012)	−0.054 (0.997)	0.232
Factor 3: meat-fish	−0.025 (0.995)	0.056 (0.998)	0.183

Mean (SD) or Median (IQR) for continuous variables, number of subjects and percentage (%) for categorical variables were presented

*Abbreviation*: *SO* sarcopenic obesity, *CSI-D* Community Screening Instrument of Dementia, *CVD* cardiovascular diseases, *PASE* physical activity scale for the elderly, *BMI* body mass index, *WC* waist circumference, *BPF* body fat percentage, *ASMI* appendicular skeletal muscle mass index, *hs-CRP* high-sensitivity C-reactive protein, *eGFR* estimated glomerular filtration rate, *DQI-I* Diet Quality Index-International, *DII* dietary inflammatory index, *MDS* the Mediterranean diet score, *DASH* the Dietary Approaches to Stop Hypertension, *MIND* the Mediterranean-DASH Intervention for Neurodegenerative Delay Diet

### Dietary patterns and incident sarcopenic obesity

**Fig. 1:**
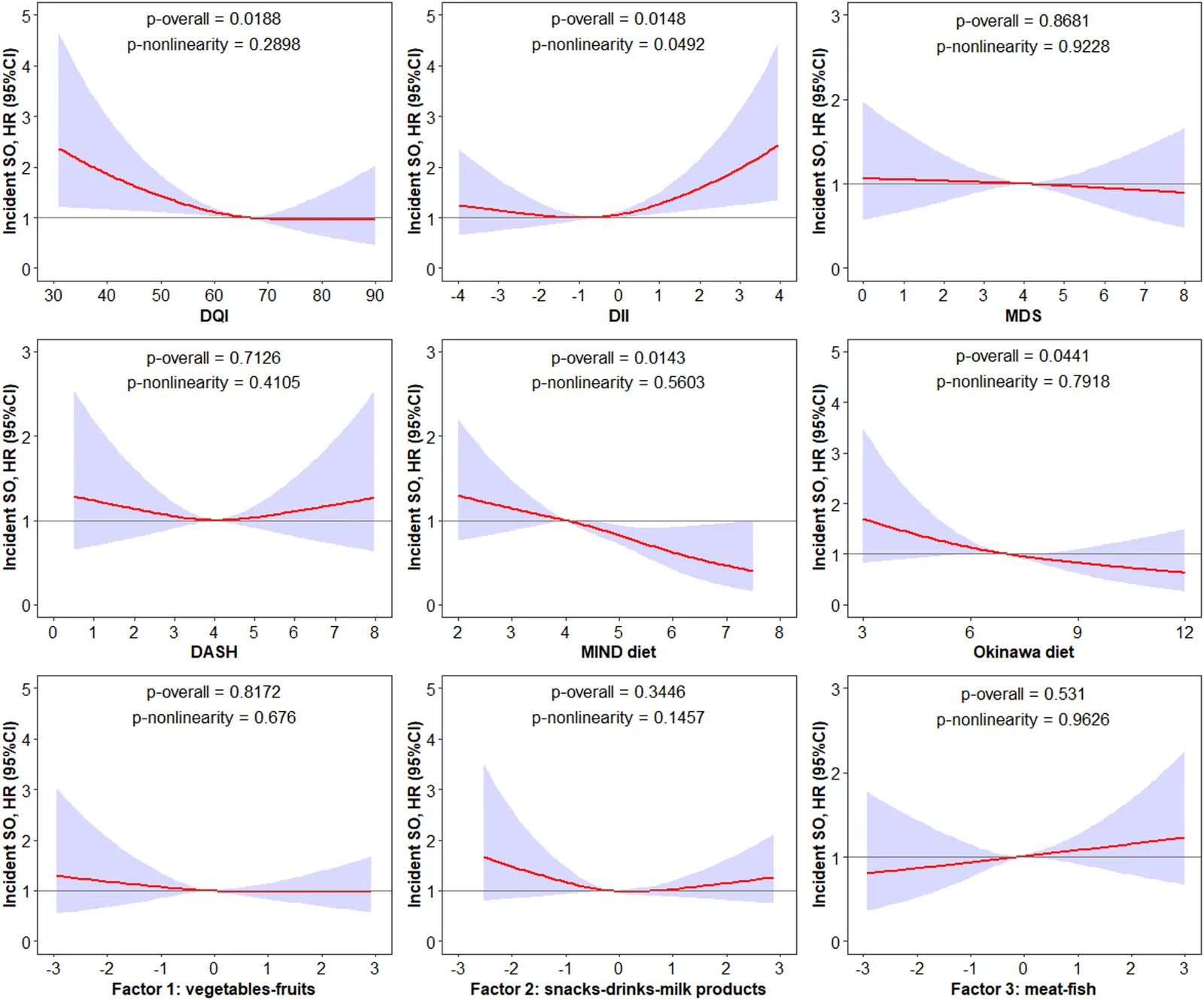
Association between dietary pattern scores and incident sarcopenic obesity by restricted cubic spline Cox regression analysis. Three knots were placed at the 10th, 50th, and 90th percentiles of the dietary pattern scores, with the median score used as the reference point. Adjusted for age, sex, educational level, living alone, smoking status, alcohol consumption, physical activity level, total energy intake, medical history of hypertension, diabetes and heart diseases, and probable dementia in Cox regression model. Abbreviation: SO, sarcopenic obesity; HR, hazard ratio; CI, confidence interval; DQI-I, Diet Quality Index-International; DII, dietary inflammatory index; MDS, the Mediterranean diet score; DASH, the Dietary Approaches to Stop Hypertension; MIND, the Mediterranean-DASH Intervention for Neurodegenerative Delay Diet

The association between dietary patterns and incident SO is presented in Table 2; Fig. 1. During four years of follow-up, 266 incident cases of SO were identified among 3,398 participants. In the age- and sex-adjusted model, higher scores for DQI-I, MIND diet, and Okinawa diet were associated with a lower risk of incident SO. The HRs per SD increase were 0.829 (95% CI 0.738–0.931) for DQI-I, 0.827 (95% CI 0.733–0.932) for MIND diet, and 0.841 (95% CI 0.746–0.949) for Okinawa diet. Moreover, higher DII scores significantly increased the risk of incident SO (HR 1.186, 95% CI 1.052–1.337). After adjustment for baseline sociodemographic factors, lifestyle factors, and medical history, the associations between these dietary scores and incident SO were slightly attenuated but remained significant. The adjusted HRs per SD increase were 0.854 (95% CI 0.756–0.964) for DQI-I, 0.853 (95% CI 0.754–0.965) for MIND diet, 0.866 (95% CI 0.764–0.982) for Okinawa diet, and 1.158 (95% CI 1.007–1.332) for DII. However, no significant associations were found between the MDS, DASH, “vegetables-fruits,” “snacks-drinks-milk products,” or “meat-fish” dietary patterns and incident SO. RCS analysis showed similar results, indicating generally linear relationships between DQI-I, DII, MIND, and Okinawa diet scores and the risk of SO. The proportional hazards assumption was verified using Schoenfeld residuals, and no violations were observed (Supplementary Table S5). After adjusting for multiple testing using the Benjamini-Hochberg FDR procedure (Supplementary Table S6), the associations of incident SO with DQI-I and MIND diet remained statistically significant. The associations for DII and Okinawa diet were attenuated to borderline significance.

**Table 2:** Association between per SD increase in dietary pattern scores and incident sarcopenic obesity risk

	Model 1		Model 2	
	HR (95% CI)	*p*	HR (95% CI)	*p*
DQI-I	0.829 (0.738–0.931)	0.002	0.854 (0.756–0.964)	0.011
DII	1.186 (1.052–1.337)	0.005	1.158 (1.007–1.332)	0.040
MDS	0.935 (0.829–1.053)	0.268	0.967 (0.854–1.094)	0.598
DASH	0.991 (0.877–1.120)	0.885	0.999 (0.884–1.131)	0.999
MIND diet	0.827 (0.733–0.932)	0.003	0.853 (0.754–0.965)	0.011
Okinawa diet	0.841 (0.746–0.949)	0.005	0.866 (0.764–0.982)	0.025
*Hong Kong diet pattern*				
Factor 1: vegetables-fruits	0.951 (0.839–1.077)	0.428	0.973 (0.857–1.103)	0.667
Factor 2: snacks-drinks-milk products	0.938 (0.825–1.066)	0.329	0.989 (0.863–1.134)	0.877
Factor 3: meat-fish	1.097 (0.976–1.234)	0.119	1.082 (0.959–1.221)	0.201

*Abbreviation*: *SD* standard deviation, *HR* hazard ratio, *CI* confidence interval, *DQI-I* Diet Quality Index-International, *DII* dietary inflammatory index, *MDS* the Mediterranean diet score, *DASH* the Dietary Approaches to Stop Hypertension, *MIND* the Mediterranean-DASH Intervention for Neurodegenerative Delay Diet

Model 1: adjusted for age and sex

Model 2: adjusted for Model 1 plus educational level, living alone, smoking status, alcohol consumption, total energy intake, physical activity level, medical history of hypertension, diabetes and heart diseases, and probable dementia

### Subgroup analysis

Of the 266 incident SO cases identified over the 4-year follow-up, 40 participants transitioned from normal status to SO, 12 from sarcopenia to SO, and 214 from obesity to SO. The incidence rates of SO varied across baseline subgroups, with the highest rate observed in the baseline obesity only group (37.28 per 1,000 person-years), compared to the normal (6.26) and sarcopenia only (31.28) groups (Table 3). Among participants with obesity at baseline, higher DQI-I, MIND, and Okinawa diet scores were significantly associated with a lower risk of developing SO (DQI-I: HR 0.866, 95% CI 0.752–0.996; MIND: HR 0.835, 95% CI 0.728–0.959; Okinawa: HR 0.858, 95% CI 0.747–0.984). In addition, higher DII scores significantly increased the risk of incident SO in this subgroup (HR: 1.223, 95% CI 1.046–1.429). However, no significant associations were observed between any dietary patterns and incident SO regarding transitions from normal status or sarcopenia only to SO. However, interaction tests showed no significant heterogeneity in the associations between dietary patterns and incident SO across different baseline body composition groups (all *p*-interactions > 0.05). In the sex-stratified analyses (Supplementary Table S7 and Table S8), higher scores for the DQI-I, MIND, and Okinawa diets were significantly associated with a lower risk of SO in women, whereas a higher DII score was associated with an increased risk of SO in men. The interaction between sex and dietary patterns were not statistically significant (all *p*-interactions > 0.05).

**Table 3:** Association between per SD increase in dietary pattern scores and incident sarcopenic obesity risk according to baseline sarcopenia and obesity status

	From normal to SO		From sarcopenia to SO		From obesity to SO	
	HR (95% CI)	*p*	HR (95% CI)	*p*	HR (95% CI)	*p*
No. of incident SO / Total	40 / 1,688		12 / 111		214 / 1,599	
Incidence rate (per 1,000 person-years)	6.26		31.28		37.28	
DQI-I	0.823 (0.602–1.124)	0.221	0.774 (0.391–1.532)	0.463	0.866 (0.752–0.996)	0.044
DII	1.024 (0.699–1.499)	0.902	0.916 (0.341–2.461)	0.861	1.223 (1.046–1.429)	0.011
MDS	0.865 (0.634–1.181)	0.362	1.227 (0.536–2.804)	0.628	0.989 (0.858–1.140)	0.881
DASH	1.130 (0.825–1.547)	0.445	1.550 (0.750–3.203)	0.236	0.977 (0.851–1.121)	0.742
MIND diet	0.856 (0.617–1.188)	0.354	0.949 (0.335–2.689)	0.921	0.835 (0.728–0.959)	0.011
Okinawa diet	0.834 (0.595–1.169)	0.293	0.860 (0.317–2.330)	0.767	0.858 (0.747–0.984)	0.029
*Hong Kong diet pattern*						
Factor 1: vegetables-fruits	1.001 (0.717–1.398)	0.995	1.272 (0.650–2.487)	0.482	0.968 (0.837–1.118)	0.658
Factor 2: snacks-drinks-milk products	1.090 (0.779–1.525)	0.614	0.897 (0.409–1.966)	0.785	0.964 (0.829–1.121)	0.634
Factor 3: meat-fish	1.115 (0.812–1.539)	0.501	0.786 (0.359–1.721)	0.547	1.048 (0.916–1.200)	0.491

*Abbreviation*: *SD* standard deviation, *HR* hazard ratio, *CI* confidence interval, *DQI-I* Diet Quality Index-International, *DII* dietary inflammatory index, *MDS* the Mediterranean diet score, *DASH* the Dietary Approaches to Stop Hypertension, *MIND* the Mediterranean-DASH Intervention for Neurodegenerative Delay Diet

Adjusted for age, sex, educational level, living alone, smoking status, alcohol consumption, physical activity level, total energy intake, medical history of hypertension, diabetes and heart diseases, and probable dementia in Cox regression model. *p*-interactions of dietary pattern scores with baseline sarcopenia and obesity status in relation to incident SO were not significant (all *p*-interactions > 0.05)

### Sensitivity analysis

In the 2-year restricted sensitivity analysis, the associations were no longer statistically significant, which may be due to limited statistical power of reduced cases (177 incident SO cases). However, the point estimates remained consistent with the primary analysis (Supplementary Table S9 and Table S10). Accounting for informative censoring using IPCW, the associations between dietary patterns and incident SO remained unchanged (Supplementary Tables S11 and Table S12). In addition, the E-values for the point estimates of the significant dietary patterns ranged from 1.58 to 1.62, which may indicate that the observed associations could be explained by a moderately strong unmeasured confounder [49, 50]. In sensitivity analyses for the competing risk of death using Fine-Gray models, the SHRs for the associations between dietary patterns and incident SO remained consistent (Supplementary Table S13 and Table S14). In the sensitivity analyses applying alternative obesity definitions (Supplementary Table S15), the associations of higher DQI and MIND diet scores with a reduced risk of incident SO, and a lower risk of developing SO from baseline obesity, remained robust and statistically significant (Supplementary Tables S16 and S17). However, the associations between the DII and Okinawa diet with SO were attenuated and lost statistical significance. When AWGS 2019-defined incident SO was used as the outcome, the associations between dietary patterns and incident SO were attenuated and no longer statistically significant (Supplementary Figure S2, Table S16 and Table S17).

After additional adjustment for serum hs-CRP, the association between per SD increase in DQI-I and incident SO was attenuated and became non-significant (HR 0.882, 95% CI 0.762, 1.022; *p* = 0.094) (Supplementary Table S18). Similarly, after additional adjustment for eGFR, the association between per SD increase in DII and incident SO was attenuated and became non-significant (HR 1.138, 95% CI 0.972, 1.333; *p* = 0.108). The associations between other dietary patterns and incident SO were not appreciably altered in the sensitivity analyses. Similar results were observed regarding the association between dietary patterns and incident SO among participants with baseline obesity (Supplementary Table S19). Subgroup analysis by serum hs-CRP or eGFR was shown in Supplementary Figure S3. There was a significant interaction between DII and eGFR in relation to incident SO (*p*-interaction < 0.001). Among individuals with eGFR levels below the median, higher DII scores were associated with an increased risk of incident SO; however, no such association was found for individuals with eGFR levels at or above the median. Although no significant interaction was detected between the “meat-fish” dietary pattern and eGFR, greater adherence to this pattern was associated with a higher risk of incident SO among those with eGFR ≥median, but no association was found for eGFR < median. No significant interactions were observed between other dietary patterns with either serum hs-CRP or eGFR. The associations between each dietary pattern score and log-transformed serum hs-CRP levels or eGFR are presented in Supplementary Table S20.

## Discussion

In this prospective cohort study of Chinese community-dwelling older adults, higher diet quality and greater adherence to an anti-inflammatory diet, MIND diet and Okinawa diet were associated with lower odds of incident SO over four-year follow-up. Our analyses observed that over 80% of incident SO were from baseline obesity. Furthermore, the protective associations of these healthy dietary patterns on incident SO were found in the progression from baseline obesity to SO, with no significant associations observed for transitions from normal or sarcopenia only to SO. Preventive efforts against SO may prioritize individuals with obesity to prevent their progression to this condition during aging. This preventive strategy could be implemented by promoting anti-inflammatory dietary patterns (lower DII scores) and antioxidant dietary patterns (greater adherence to MIND diet and Okinawa diet).

Our findings are consistent with previous studies reporting that DASH diet, Mediterranean diet, or other healthy dietary patterns rich in vegetables, fruits, whole grains, fish and seafood, were associated with a reduced risk of SO in several cross-sectional studies [13, 16, 17, 51]. In addition, pro-inflammatory diets and diets with lower composite antioxidant capacity were associated with an increased risk of SO [14, 52]. In this prospective study, we observed that higher overall diet quality, lower DII scores, and greater adherence to the MIND and Okinawa diets were protective against SO. These healthy dietary patterns share common characteristics, including higher consumption of whole grains, vegetables, fruits, soy and soy products, and legumes, and lower consumption of refined grains, red and processed meats, fat and oil. The anti-inflammatory and antioxidant components in these diets may attenuate inflammation and reduce lipid peroxidation and oxidative DNA damage, thereby reducing muscle catabolism and adipose tissue accumulation [19]. In addition, their higher dietary fiber content might modulate gut microbiota by increasing the abundance of beneficial bacteria; this could potentially promote metabolic health through mechanisms such as increasing the production of short-chain fatty acids, which are hypothesized to help alleviate inflammation and improve insulin sensitivity [53]. Given that SO is characterized by a state of chronic low-grade inflammation, these anti-inflammatory and antioxidative properties are likely key protective mechanisms [3]. Supporting this mechanistic link, previous studies have reported that biomarkers of systemic inflammation (e.g., C-reactive protein-albumin-lymphocyte index and the neutrophil-percentage-to-albumin ratio) mediated associations between pro-inflammatory and low-antioxidant diets and SO risk [14, 52]. Importantly, the protective associations of the DQI-I and MIND diets with incident SO remained robust after FDR adjustment and across alternative obesity definitions, suggesting their benefit for older adults with pre-existing obesity. Associations for the DII and Okinawa diets lost significance under these alternative criteria, warranting cautious interpretation. When the AWGS 2019 criteria was used to define SO, the associations between dietary patterns and incident SO were attenuated and no longer statistically significant. This may reflect differences in the phenotypes captured by the AWGS 2019 and AWGS 2025 algorithms. AWGS 2019 incorporates physical performance, including gait speed and five-time chair stand test, which may be influenced by non-nutritional factors such as physical activity, osteoarthritis, pain, neurological conditions, balance problems, and cardiopulmonary limitations. In contrast, the AWGS 2025 definition used in our primary analysis is based on low muscle mass, low muscle strength, and obesity. Its consideration of BMI-adjusted muscle mass indices (ASM/BMI) may capture a more body-composition-oriented SO phenotype that is more closely related to dietary exposures. Future studies are needed to further compare SO definitions across populations and measurement settings.

Although adherence to the MIND and Okinawa diets protected against incident SO, the *a posteriori* “vegetables-fruits” pattern showed null results. This discrepancy may stem from fundamental methodological differences. Comprehensive *a priori* indices (MIND and Okinawa diet) recommend specific plant foods (e.g., berries, green leafy vegetables) while limiting pro-inflammatory components (e.g., red meats, fast food, sweets). In contrast, our empirically derived “vegetables-fruits” factor reflects the co-consumption of plant-based foods. Because its factor loadings for pro-inflammatory items, such as sweets, fast food, and red and processed meats, were near zero, a high score on this pattern does not preclude concurrent high consumption of these detrimental foods. Therefore, the isolated benefits of vegetables and fruits may be offset by uncaptured pro-inflammatory intakes, rendering the pattern insufficient to mitigate SO risk. Notably, we also did not find statistically significant associations for the DASH or Mediterranean dietary patterns. This discrepancy may be attributed to the fact that these Western-origin diets do not fully align with traditional Chinese dietary habits. For instance, in our study population of Chinese older adults, fewer than 20% of participants reached a score indicative of high adherence to the Mediterranean diet (> 6 out of 9) [20], which may have reduced variability in exposure and limited statistical power to detect associations.

In the context of aging, individuals with obesity are particularly susceptible to SO through multiple pathways, including age-related hormonal decline, anabolic resistance, mitochondrial dysfunction, insulin resistance and inflammaging, which collectively accelerate muscle mass loss and impair physical performance [3, 5, 54]. Consistent with this framework, most incident SO cases in our cohort arose from older adults with pre-existing obesity, with approximately 80% of incident SO developing among participants with baseline obesity. In obesity, the excess of android and visceral fat promotes systemic inflammation and can lead to ectopic lipid deposition within skeletal muscle. Accumulated intramuscular lipids and lipotoxic intermediates may impair mitochondrial function, leading to reduced β-oxidation capacity and increased reactive oxygen species production [3, 55]. These metabolic disturbances can, in turn, trigger local inflammatory signaling and alter myokine secretion, which may impair muscle function. The dysregulated myokines may further aggravate adipose tissue inflammation and sustain chronic low-grade systemic inflammation, reinforcing a vicious cycle between adiposity and muscle dysfunction [3, 55]. Against this background, our findings suggest that higher diet quality may be particularly relevant for preventing progression from obesity to SO. Specifically, greater adherence to anti-inflammatory (lower DII scores) and antioxidative (higher MIND and Okinawa diet scores) dietary patterns were associated with a lower risk of incident SO among participants with baseline obesity. In contrast, no significant associations were observed for transitions from normal status or sarcopenia alone to SO. Because only approximately 20% of incident SO cases originated from these baseline states, the reduced sample size in these specific transition pathways limited our statistical power to detect potential associations. Therefore, the lack of significant findings in these subgroups should be interpreted with caution, and future large-scale cohorts are needed to further investigate these specific transitions. Nevertheless, given that the majority of incident SO cases in our cohort originated from baseline obesity, this finding may suggest that dietary interventions aimed at preventing SO should primarily target and prioritize older adults with pre-existing obesity.

The sex-stratified analyses suggested potentially sex-specific associations, although the interaction terms were not statistically significant. We observed that healthy dietary patterns (DQI-I, MIND, and Okinawa diets) showed significant protective associations primarily in women, whereas the pro-inflammatory diet (DII) was significantly associated with increased SO risk in men. Men are predisposed to accumulate visceral fat, which is highly pro-inflammatory, potentially increasing their susceptibility to the adverse effects of an inflammatory diet [56]. Conversely, women, especially post-menopausal women facing estrogen decline, may benefit more from the synergistic effects of overall nutrient density provided by high-quality diets to mitigate muscle loss and fat infiltration [57]. Given the lack of significant statistical interaction, these sex-specific findings should be interpreted with caution and warrant further investigation in larger cohorts.

The inverse association between DQI-I and incident SO was attenuated after additional adjustment for serum hs-CRP in sensitivity analyses. This attenuation suggests that overall diet quality and systemic inflammation may share overlapping variance in their relationship with SO risk. A similar finding has been reported in a cross-sectional analysis of the PREDIMED-Plus study (1,535 Spanish adults with overweight/obesity or metabolic syndrome), where additional adjustment for potential covariates, including inflammatory markers, diabetes, and waist circumference, dietary variables lost significance associated with SO risk [13]. These findings may indicate the interconnected nature of diet, systemic inflammation, and metabolic health. However, in our analysis, the associations for DII, and adherence to the MIND or Okinawa diets were not materially attenuated after adjusting for serum hs-CRP. A potential explanation is that hs-CRP, while widely used, may not fully characterize the complexity of immune system and inflammatory pathway [45]. In particular, MIND and Okinawa diets may act not only through inflammation but also through other biological processes, such as oxidative stress, mitochondrial function, or metabolic regulation [58]. Future studies incorporating a broader panel of inflammatory and oxidative stress biomarkers are needed to better examine the interrelationships between dietary patterns and the development of SO.

A significant interaction between DII and eGFR on the development of SO was observed in this study. The association between a pro-inflammatory diet (higher DII scores) and incident SO was more evident among participants with lower renal function (eGFR < median). Although eGFR is primarily an indicator of renal function, reduced eGFR is often accompanied by chronic low-grade inflammation, which may increase the risk of muscle catabolism and metabolic dysregulation [59]. The adverse associations of pro-inflammatory diets may be manifested among individuals with impaired renal function due to an already elevated inflammatory milieu or greater comorbidity burden [59, 60]. We also found that greater adherence to the “meat-fish” dietary pattern was associated with a higher risk of SO among those with preserved renal function (eGFR ≥median). This may suggest that renal function may confound the association between animal-based dietary patterns and SO risk, potentially because individuals with lower eGFR may reduce meat intake or follow specific dietary restrictions [61]. While adequate protein is important for maintaining muscle mass, higher intakes of animal protein, potentially accompanied by saturated fat, heme iron, sodium, and specific cooking methods, may be associated with adverse metabolic or inflammatory profiles [62]. In addition, higher protein intake may increase renal burden and impair renal function, especially in older adults [61]. Future studies are needed to clarify the optimal protein source and amount for SO prevention, while accounting for renal function and inflammation in older population [9].

This study has several strengths. First, this study examined whether associations between dietary patterns and incident SO differed by baseline body composition status, thereby distinguishing the role of diet in trajectories from obesity to SO and from sarcopenia to SO. In addition, it had a relatively large sample of community-dwelling older Chinese adults. validated dietary assessment tools were used to characterize habitual dietary patterns, and DXA scan was used to quantify muscle and fat mass. A wide range of potential confounders were also considered, including sociodemographic characteristics, lifestyle behaviors, and inflammatory biomarkers. However, several limitations should be acknowledged. First, the relatively short follow-up duration may have a limited number of incident SO cases. The statistical power to detect associations in the transition from sarcopenia only to SO or from normal to SO was limited due to the small number of events. Future studies with larger sample sizes and longer follow-up periods are warranted to adequately power these specific transition analyses. Second, dietary intake was assessed only at baseline, limiting our ability to examine changes in diets over time and their potential impact on SO risk. Although previous studies suggest that dietary patterns in older adults are relatively stable over a 4-year period [63, 64], changes in diet during follow-up may introduce non-differential exposure misclassification. This may bias the estimates toward the null, suggesting that the true associations might be stronger than observed. This is supported by our 2-year sensitivity analysis, which showed consistent point estimates despite a loss of statistical significance due to reduced power. Third, applying the Okinawa diet score to this Hong Kong cohort resulted in all participants scoring zero for the sweet potato component. This lack of variance restricted the score range, potentially attenuating the observed protective associations. Fourth, inflammatory or oxidative stress biomarkers beyond hs-CRP were not measured in this study. Future research is needed to consider incorporating a broader range of biomarkers to provide a more comprehensive understanding of the underlying mechanisms involved in the development of SO. Finally, the participants were volunteers who may have had higher educational attainment, greater health awareness, and higher physical activity than the general older adult population in Hong Kong. In addition, participants who lost to follow-up tended to be older and in poorer health than those who completed the study. However, our sensitivity analyses using IPCW showed consistent results, suggesting that the observed differential attrition did not substantially bias our main findings. Nevertheless, these factors may introduce healthy cohort and attrition bias, which could limit the generalizability of the findings.

## Conclusion

This prospective cohort study suggests that higher overall diet quality and greater adherence to dietary patterns with anti-inflammatory (lower DII scores) and antioxidant features (greater adherence to the MIND and Okinawa diets) were associated with a lower risk of incident SO. Given that the progression to SO predominantly occurs in those with pre-existing obesity, dietary improvement may be particularly relevant for preventing progression from obesity to SO during aging. Future large-scale longitudinal studies and interventional studies are needed to validate these associations, explore underlying mechanisms, and inform precision nutrition strategies tailored to SO prevention according to baseline body composition status.

## Supplementary Information

Supplementary Material 1.

## Data Availability

The data that support the findings of this study are available from the corresponding author upon reasonable request.

## Abbreviations

ASMI: appendicular skeletal muscle mass index
AWGS: Asian Working Group for Sarcopenia
BFP: body fat percentage
BMI: body mass index
CI: confidence interval
CSI-D: Community Screening Instrument of Dementia
CVD: cardiovascular diseases
DASH: the Dietary Approaches to Stop Hypertension
DII: dietary inflammatory index
DQI-I: Diet Quality Index-International
DXA: dual-energy X-ray absorptiometry
eGFR: estimated glomerular filtration rate
FDR: False Discovery Rate
FFQ: food frequency questionnaire
HR: hazard ratio
hs-CRP: high-sensitivity C-reactive protein
IPCW: Inverse Probability of Censoring Weighting
IQR: interquartile range
MDS: the Mediterranean diet score
MIND: the Mediterranean-DASH Intervention for Neurodegenerative Delay Diet
PASE: physical activity scale for the elderly
RCS: restricted cubic spline
SD: standard deviation
SHR: subdistribution hazard ratio
SO: sarcopenic obesity
WC: waist circumference
