# Supplementary material for: Dietary patterns and the progression from obesity to sarcopenic obesity in older adults: a prospective cohort study
Source: BMC Geriatr. 2026 Jun 25;26:1156. doi: 10.1186/s12877-026-07857-1 (PMC13563728; doi:10.1186/s12877-026-07857-1)
Supplement: Supplementary file 1 [file 12877_2026_7857_MOESM1_ESM.docx]

**Supplementary materials**

**List of supplementary materials**

| **Contents** | **Page** |
| --- | --- |
| **Figure S1**. Flowchart of the study participants. | 3 |
| **Table S1.** Detailed components of *a priori* dietary patterns in this study. | 4-5 |
| **Table S2.** Food group factor loading for *a posterior* dietary pattern. | 6 |
| **Table S3**. Baseline characteristics of study participants according to baseline body composition status. | 7-8 |
| **Table S4.** Baseline characteristics of participants included and participants loss of follow-up. | 9-10 |
| **Table S5.** Proportional hazards assumption check for dietary patterns using Schoenfeld residuals. | 11 |
| **Table S6.** Association between per SD increase in dietary pattern scores and incident sarcopenic obesity risk with FDR adjustment. | 12 |
| **Table S7.** Sex-specific association between per SD increase in dietary pattern scores and incident sarcopenic obesity risk. | 13 |
| **Table S8.** Sex-specific association between per SD increase in dietary pattern scores and incident sarcopenic obesity risk among individuals with baseline obesity. | 14 |
| **Table S9.** Sensitivity analysis for association between per SD increase in dietary pattern scores and incident sarcopenic obesity risk over 2 years of follow-up. | 15 |
| **Table S10.** Sensitivity analysis for association between per SD increase in dietary pattern scores and incident sarcopenic obesity risk among individuals with baseline obesity over 2 years of follow-up. | 16 |
| **Table S11.** Association between per SD increase in dietary pattern scores and incident sarcopenic obesity risk before and after IPCW. | 17 |
| **Table S12.** Association between per SD increase in dietary pattern scores and incident sarcopenic obesity risk among individuals with baseline obesity before and after IPCW. | 18 |
| **Table S13.** Sensitivity analysis for association between per SD increase in dietary pattern scores and incident sarcopenic obesity risk using Fine-Gray competing risk model. | 19 |
| **Table S14.** Sensitivity analysis for association between per SD increase in dietary pattern scores and incident sarcopenic obesity risk among individuals with baseline obesity using Fine-Gray competing risk model. | 20 |
| **Table S15**. Different criteria for sarcopenia and obesity. | 21 |
| **Figure S2**. Venn diagram showing the overlap of incident SO cases defined by AWGS 2019 or AWGS 2025 criteria. | 22 |
| **Table S16.** Sensitivity analysis for association between per SD increase in dietary pattern scores and incident sarcopenic obesity risk based on different categories of sarcopenia and obesity. | 23-24 |
| **Table S17.** Sensitivity analysis for association between per SD increase in dietary pattern scores and incident sarcopenic obesity risk among individuals with baseline obesity based on different categories of sarcopenia and obesity. | 25-26 |
| **Table S18.** Sensitivity analysis for association between per SD increase in dietary pattern scores and incident sarcopenic obesity risk. | 27 |
| **Table S19.** Sensitivity analysis for the association between per SD increase in dietary pattern scores and incident sarcopenic obesity risk among individuals with baseline obesity. | 28 |
| **Figure S3.** Subgroup analysis for association between per SD increase in dietary pattern scores and incident sarcopenic obesity risk based on the median of serum hs-CRP or eGFR. | 29 |
| **Table S20.** Associations between each dietary pattern score with log-transformed serum hs-CRP levels and eGFR in multivariable adjusted linear regression model. | 30 |

**Figure S1.** The flowchart of study participants.

**Table S1**. Detailed components of *a priori* dietary patterns in this study.

| **DQI-I** | **DII** | **MDS** | **DASH** | **MIND** | **Okinawan** |
| --- | --- | --- | --- | --- | --- |
| **Range: 0-94** | **Range: −4.66, 5.13** | **Range: 0-9** | **Range: 0-9** | **Range: 0-15** | **Range: 0-16** |
| **Variety:**  Overall food group variety (meat/poultry/fish/eggs/ dairy/beans; grain; fruit; vegetable)  Within-group variety for protein source (meat, poultry, fish, dairy, beans, eggs)  **Adequacy:**  Vegetable group,  Fruit group,  Grain group,  Fiber,  Protein,  Iron,  Calcium,  Vitamin C  **Overall balance:**  Macronutrient ratio (carbohydrate:protein:fat),  Fatty acid ratio (PUFA:MUFA:SFA) | **Anti-inflammatory components:**  Protein,  Monounsaturated fatty acid,  Polyunsaturated fatty acid,  Vitamin A,  b-carotene,  Thiamin,  Riboflavin,  Niacin,  Vitamin B6,  Folic acid,  Vitamin C,  Vitamin D,  Vitamin E,  Magnesium,  Selenium,  Zinc,  Fiber,  Isoflavones,  Caffeine,  Onion,  Pepper,  Green/black tea | **Beneficial components:**  Vegetables, Legumes/seeds/nuts,  Fruits,  Cereals,  Fish,  Monounsaturated to saturated lipid ratio | **Beneficial components:**  Potassium, Magnesium,  Calcium,  Protein,  Fiber | **Beneficial components:**  Green leafy vegetables,  Other vegetables, Nuts,  Berries,  Legumes,  Whole grains, Seafood,  Poultry,  Olive oil,  Wine | The corresponding percentage of total energy intake:  Sweet potatoes (69%),  Rice (12%),  Other grains including wheat and Barley (7%),  Legumes (6%),  Vegetables (3%),  Oils (2%),  Fish (1%) |
| **Moderate:**  Total fat,  Saturated fat,  Cholesterol,  Sodium |  | **Moderate components:**  Ethanol | **Moderate components:**  Total fat |  |  |
|  | **Pro-inflammatory components:**  Carbohydrate,  Total fat,  Saturated fat,  Cholesterol,  Vitamin B12,  Iron | **Detrimental components:**  Meat,  Poultry,  Dairy products | **Detrimental components:**  Sodium,  Saturated fat,  Cholesterol | **Detrimental components:**  Red meat  Butter and margarine,  Cheese,  Pastries and sweets,  Fried fast food | <1% each for nuts and seeds, meat, eggs, dairy, fruit, other potatoes, sugars, pickled vegetables, flavors, and alcohol |

Abbreviation: DQI-I, Diet Quality Index-International; DII, dietary inflammatory index; MDS, the Mediterranean diet score; DASH, the Dietary Approaches to Stop Hypertension; MIND, the Mediterranean-DASH Intervention for Neurodegenerative Delay Diet; Polyunsaturated fatty acid, PUFA; Monounsaturated fatty acid, MUFA; Saturated fatty acid, SFA.

Higher dietary pattern scores indicated greater adherence to the dietary pattern.

**Table S2**. Food group factor loading for *a posterior* dietary pattern. ^a^

|  | **Dietary pattern** | | |
| --- | --- | --- | --- |
| **Food group** | **Factor 1:**  **Vegetables-fruits** | **Factor 2:**  **snacks-drinks-dairy products** | **Factor 3:**  **meat-fish** |
| Other vegetables | **0.58** | −0.06 | 0.02 |
| Tomatoes | **0.49** | 0.03 | −0.01 |
| Dark green and leafy vegetables | **0.43** | −0.26 | −0.02 |
| Cruciferous vegetables | **0.43** | −0.05 | −0.06 |
| Starchy vegetables | **0.42** | 0.03 | 0.00 |
| Soy and soy products | **0.42** | 0.08 | 0.11 |
| Fruits | **0.40** | 0.03 | −0.01 |
| Legumes | **0.34** | −0.01 | 0.02 |
| Mushrooms and fungi | **0.22** | 0.06 | −0.07 |
| Fats and oils | **−0.37** | −0.21 | 0.15 |
| Condiments | −0.05 | **0.48** | −0.13 |
| Coffee | −0.15 | **0.42** | −0.17 |
| Fast food | −0.03 | **0.37** | 0.04 |
| Nuts | 0.12 | **0.37** | −0.03 |
| French fries and potato chips | −0.03 | **0.37** | 0.09 |
| Milk and milk products | 0.08 | **0.31** | −0.14 |
| Whole grains | 0.14 | **0.30** | −0.17 |
| Sweets and desserts | 0.02 | **0.29** | 0.08 |
| Beverages | −0.03 | **0.22** | 0.09 |
| Dim sum | −0.17 | −0.11 | **0.56** |
| Red and processed meats | −0.07 | 0.06 | **0.46** |
| Poultry | 0.06 | 0.11 | **0.45** |
| Fish and seafood | 0.22 | −0.17 | **0.37** |
| Wine | −0.14 | 0.10 | **0.20** |
| Refined grains | −0.25 | −0.50 | **−0.69** |
| Cakes, cookies, pies and biscuits | 0.06 | 0.14 | 0.19 |
| Eggs | 0.07 | 0.19 | 0.05 |
| Organ meats | −0.08 | 0.15 | 0.12 |
| Others | 0.01 | 0.07 | 0.03 |
| Preserved vegetables | −0.02 | 0.08 | 0.00 |
| Soups | 0.00 | 0.00 | −0.01 |
| Tea | 0.00 | 0.15 | 0.03 |
| % Variance explained | 6.2 | 5.4 | 5.1 |

^a^ Factor loading with absolute value ≥0.2 are shown in bold. For food group loads more than one dietary pattern, the highest loading is bolded.

**Table S3.** Baseline characteristics of study participants according to baseline body composition status.

|  | **Normal** | **Sarcopenia only** | **Obesity**  **only** | ***p*** |
| --- | --- | --- | --- | --- |
| N | 1,688 | 111 | 1,599 |  |
| Age, years | 71.94 (4.98) | 76.12 (6.22) ^a^ | 71.89 (4.64) ^b^ | <0.001 |
| Women, n (%) | 847 (50.18) | 32 (22.83) ^a^ | 810 (52.10) ^b^ | <0.001 |
| Education, n (%) |  |  |  | 0.136 |
| No education | 352 (20.85) | 14 (12.61) | 332 (20.76) |  |
| Primary or below | 852 (50.47) | 68 (61.26) | 792 (49.53) |  |
| Secondary or above | 484 (28.67) | 29 (26.13) | 475 (29.71) |  |
| Living alone, n (%) | 193 (11.43) | 10 (9.01) | 153 (9.57) | 0.191 |
| Smoking status, n (%) |  |  |  | <0.001 |
| Non-smokers | 1,072 (63.51) | 53 (47.75) | 1,049 (65.60) |  |
| Former smokers | 478 (28.32) | 42 (37.84) | 482 (30.14) |  |
| Current smokers | 138 (8.18) | 16 (14.41) ^a^ | 68 (4.25) ^a, b^ |  |
| Alcohol drinking, n (%) | 243 (14.40) | 15 (13.51) | 202 (12.63) | 0.333 |
| CSI-D | 30.27 (2.01) | 30.22 (1.75) | 30.35 (1.87) | 0.433 |
| Probable dementia, n (%) | 241 (14.28) | 21 (18.92) | 208 (13.01) | 0.165 |
| CVD, n (%) | 279 (16.53) | 18 (16.22) | 383 (23.95) ^a^ | <0.001 |
| Diabetes mellitus, n (%) | 249 (14.75) | 13 (11.71) | 225 (14.07) | 0.622 |
| Hypertension, n (%) | 611 (36.20) | 31 (27.93) | 787 (49.22) ^a, b^ | <0.001 |
| PASE score | 97.77 (44.77) | 80.56 (46.10) ^a^ | 88.86 (40.49) ^a^ | <0.001 |
| Weight, kg | 55.02 (8.57) | 47.14 (6.44) ^a^ | 63.29 (8.65) ^a, b^ | <0.001 |
| BMI, kg/m^2^ | 22.13 (2.54) | 19.55 (2.45) ^a^ | 25.60 (2.74) ^a, b^ | <0.001 |
| WC, cm | 82.30 (8.17) | 76.59 (8.28) ^a^ | 91.18 (7.86) ^a, b^ | <0.001 |
| BFP, % | 25.82 (6.10) | 22.15 (6.61) ^a^ | 33.56 (5.74) ^a, b^ | <0.001 |
| ASMI, kg/m^2^ | 6.59 (0.94) | 5.96 (0.68) ^a^ | 6.77 (0.97) ^a, b^ | <0.001 |
| ASM/BMI | 0.75 (0.14) | 0.75 (0.14) | 0.66 (0.13) ^a, b^ | <0.001 |
| Grip strength, kg | 29.28 (8.17) | 21.06 (4.70) ^a^ | 28.53 (7.78) ^a, b^ | <0.001 |
| Gait speed, m/s | 1.05 (0.23) | 0.95 (0.21) ^a^ | 1.01 (0.21) ^a, b^ | <0.001 |
| Chair stand test, s | 12.62 (4.15) | 13.39 (4.40) | 13.08 (4.47) ^a^ | 0.005 |
| Serum hs-CRP, mg/L | 1.30 (0.60–2.70) | 0.90 (0.50–2.30) | 2.10 (1.10–3.90) ^a, b^ | <0.001 |
| eGFR, mL/min/1.73 m^2^ | 85.34 (74.10–90.97) | 84.35 (71.34–89.62) | 84.58 (72.02–91.29) | 0.151 |
| Energy intake, kcal/day | 1875.63 (584.20) | 1883.04 (559.26) | 1817.77 (566.20) ^a^ | 0.013 |
| Dietary pattern scores |  |  |  |  |
| DQI-I | 65.20 (9.45) | 62.51 (10.59) ^a^ | 64.40 (9.32) ^a^ | 0.002 |
| DII | −0.57 (1.48) | −0.36 (1.47) | −0.49 (1.45) | 0.154 |
| MDS | 4.12 (1.53) | 3.79 (1.48) | 4.09 (1.54) | 0.094 |
| DASH | 4.05 (1.26) | 3.72 (1.23) ^a^ | 3.97 (1.27) | 0.015 |
| MIND diet | 4.63 (0.93) | 4.32 (1.04) ^a^ | 4.61 (0.92) ^b^ | 0.003 |
| Okinawa diet | 7.22 (1.38) | 7.21 (1.48) | 7.17 (1.39) | 0.637 |
| *Hong Kong diet pattern* |  |  |  |  |
| Factor 1: vegetables-fruits | 0.031 (1.007) | −0.217 (1.199)^a^ | 0.006 (0.966) | 0.038 |
| Factor 2: snacks-drinks-milk products | 0.027 (1.000) | 0.025 (1.065) | 0.005 (1.018) | 0.810 |
| Factor 3: meat-fish | −0.068 (0.980) | 0.232 (1.069) ^a^ | 0.016 (1.002) ^a^ | 0.001 |

Mean (SD) or Median (IQR) for continuous variables, number of subjects and percentage (%) for categorical variables were presented.

Abbreviation: SO, sarcopenic obesity; CSI-D, Community Screening Instrument of Dementia; CVD, cardiovascular diseases; PASE, physical activity scale for the elderly; BMI, body mass index; WC, waist circumference; BPF, body fat percentage; ASMI, appendicular skeletal muscle mass index; hs-CRP, high-sensitivity C-reactive protein; eGFR, estimated glomerular filtration rate; DQI-I, Diet Quality Index-International; DII, dietary inflammatory index; MDS, the Mediterranean diet score; DASH, the Dietary Approaches to Stop Hypertension; MIND, the Mediterranean-DASH Intervention for Neurodegenerative Delay Diet.

^a^ *p* < 0.05 versus the normal group; ^b^ *p* < 0.05 versus the sarcopenia only group.

**Table S4.** Baseline characteristics of participants included and participants loss of follow-up.

|  | **Participants included** | **Participants loss of follow-up** | ***p*** |
| --- | --- | --- | --- |
| N | 3,398 | 410 |  |
| Age, years | 72.05 (4.93) | 74.47 (4.84) | <0.001 |
| Women, n (%) | 1,712 (50.38) | 221 (53.90) | 0.178 |
| Education, n (%) |  |  | <0.001 |
| No education | 698 (20.54) | 123 (30.00) |  |
| Primary or below | 1,712 (50.38) | 202 (49.27) |  |
| Secondary or above | 988 (29.08) | 85 (20.73) |  |
| Living alone, n (%) | 356 (10.48) | 61 (14.88) | 0.007 |
| Smoking status, n (%) |  |  | 0.079 |
| Non-smokers | 2,174 (63.98) | 263 (64.15) |  |
| Former smokers | 1,002 (29.49) | 109 (26.59) |  |
| Current smokers | 222 (6.53) | 38 (9.27) |  |
| Alcohol drinking, n (%) | 460 (13.54) | 38 (9.27) | 0.015 |
| CSI-D | 30.30 (1.94) | 29.40 (2.58) | <0.001 |
| Probable dementia, n (%) | 470 (13.83) | 103 (25.12) | <0.001 |
| CVD, n (%) | 680 (20.01) | 101 (24.63) | 0.029 |
| Diabetes mellitus, n (%) | 487 (14.33) | 69 (16.83) | 0.176 |
| Hypertension, n (%) | 1,429 (42.05) | 180 (43.90) | 0.474 |
| PASE score | 93.02 (43.12) | 84.85 (41.32) | <0.001 |
| Weight, kg | 58.65 (9.70) | 57.06 (10.77) | 0.002 |
| BMI, kg/m^2^ | 23.68 (3.23) | 23.35 (3.83) | 0.059 |
| WC, cm | 86.29 (9.31) | 86.29 (9.80) | 0.996 |
| BFP, % | 29.34 (7.19) | 29.21 (7.71) | 0.730 |
| ASMI, kg/m^2^ | 6.66 (0.96) | 6.48 (1.00) | <0.001 |
| ASM/BMI | 0.71 (0.14) | 0.69 (0.15) | 0.038 |
| Grip strength, kg | 28.66 (8.03) | 26.79 (8.13) | <0.001 |
| Gait speed, m/s | 1.03 (0.22) | 0.94 (0.24) | <0.001 |
| Chair stand test, s | 12.86 (4.31) | 13.81 (5.38) | <0.001 |
| Serum hs-CRP, mg/L | 1.60 (0.80–3.30) | 2.10 (1.00–4.50) | <0.001 |
| eGFR, mL/min/1.73 m^2^ | 84.97 (73.05–91.10) | 80.71 (66.58–92.66) | 0.925 |
| Energy intake, kcal/day | 1848.65 (575.57) | 1759.78 (574.17) | 0.003 |
| Dietary pattern scores |  |  |  |
| DQI-I | 64.74 (9.44) | 62.55 (10.02) | <0.001 |
| DII | −0.52 (1.47) | −0.12 (1.54) | <0.001 |
| MDS | 4.09 (1.53) | 4.06 (1.56) | 0.832 |
| DASH | 4.00 (1.27) | 3.87 (1.30) | 0.026 |
| MIND diet | 4.61 (0.93) | 4.41 (0.98) | <0.001 |
| Okinawa diet | 7.20 (1.39) | 7.02 (1.41) | 0.016 |
| *Hong Kong diet pattern* |  |  |  |
| Factor 1: vegetables-fruits | 0.011 (0.995) | −0.050 (1.035) | 0.239 |
| Factor 2: snacks-drinks-milk products | 0.017 (1.011) | −0.092 (0.978) | 0.039 |
| Factor 3: meat-fish | −0.018 (0.995) | 0.078 (1.004) | 0.063 |

Mean (SD) or Median (IQR) for continuous variables, number of subjects and percentage (%) for categorical variables were presented.

Abbreviation: SO, sarcopenic obesity; CSI-D, Community Screening Instrument of Dementia; CVD, cardiovascular diseases; PASE, physical activity scale for the elderly; BMI, body mass index; WC, waist circumfrerence; BPF, body fat percentage; ASMI, appendicular skeletal muscle mass index; hs-CRP, high-sensitivity C-reactive protein; eGFR, estimated glomerular filtration rate; DQI-I, Diet Quality Index-International; DII, dietary inflammatory index; MDS, the Mediterranean diet score; DASH, the Dietary Approaches to Stop Hypertension; MIND, the Mediterranean-DASH Intervention for Neurodegenerative Delay Diet.

**Table S5**. Proportional hazards assumption check for dietary patterns using Schoenfeld residuals.

|  | ***p*-value for dietary pattern** |  | **Global model *p*-value** |
| --- | --- | --- | --- |
| DQI-I | 0.4062 |  | 0.2794 |
| DII | 0.3712 |  | 0.2705 |
| MDS | 0.2256 |  | 0.2219 |
| DASH | 0.8094 |  | 0.2954 |
| MIND diet | 0.6009 |  | 0.2836 |
| Okinawa diet | 0.2593 |  | 0.2473 |
| *Hong Kong diet pattern* |  |  |  |
| Factor 1: vegetables-fruits | 0.2202 |  | 0.2231 |
| Factor 2: snacks-drinks-milk products | 0.1993 |  | 0.2140 |
| Factor 3: meat-fish | 0.4876 |  | 0.2647 |

Abbreviation: DQI-I, Diet Quality Index-International; DII, dietary inflammatory index; MDS, the Mediterranean diet score; DASH, the Dietary Approaches to Stop Hypertension; MIND, the Mediterranean-DASH Intervention for Neurodegenerative Delay Diet.

The proportional hazards assumption was tested using Schoenfeld residuals based on the fully adjusted Cox proportional hazards models. A *p*-value >0.05 indicates that the proportional hazards assumption is not violated.

**Table S6.** Association between per SD increase in dietary pattern scores and incident sarcopenic obesity risk with FDR adjustment.

|  | **Model 1** | | |  | **Model 2** | | |
| --- | --- | --- | --- | --- | --- | --- | --- |
|  | **HR (95% CI)** | ***p*** | **FDR-adjusted *p*** |  | **HR (95% CI)** | ***p*** | **FDR-adjusted *p*** |
| DQI-I | 0.829 (0.738–0.931) | 0.002 | 0.011 |  | 0.854 (0.756–0.964) | 0.011 | 0.049 |
| DII | 1.186 (1.052–1.337) | 0.005 | 0.011 |  | 1.158 (1.007–1.332) | 0.040 | 0.090 |
| MDS | 0.935 (0.829–1.053) | 0.268 | 0.402 |  | 0.967 (0.854–1.094) | 0.598 | 0.857 |
| DASH | 0.991 (0.877–1.120) | 0.885 | 0.885 |  | 0.999 (0.884–1.131) | 0.999 | 0.999 |
| MIND diet | 0.827 (0.733–0.932) | 0.003 | 0.011 |  | 0.853 (0.754–0.965) | 0.011 | 0.049 |
| Okinawa diet | 0.841 (0.746–0.949) | 0.005 | 0.011 |  | 0.866 (0.764–0.982) | 0.025 | 0.075 |
| *Hong Kong diet pattern* |  |  |  |  |  |  |  |
| Factor 1: vegetables-fruits | 0.951 (0.839–1.077) | 0.428 | 0.481 |  | 0.973 (0.857–1.103) | 0.667 | 0.857 |
| Factor 2: snacks-drinks-milk products | 0.938 (0.825–1.066) | 0.329 | 0.423 |  | 0.989 (0.863–1.134) | 0.877 | 0.987 |
| Factor 3: meat-fish | 1.097 (0.976–1.234) | 0.119 | 0.214 |  | 1.082 (0.959–1.221) | 0.201 | 0.362 |

Abbreviation: SD, standard deviation; HR, hazard ratio; CI, confidence interval; DQI-I, Diet Quality Index-International; DII, dietary inflammatory index; MDS, the Mediterranean diet score; DASH, the Dietary Approaches to Stop Hypertension; MIND, the Mediterranean-DASH Intervention for Neurodegenerative Delay Diet.

Model 1: adjusted for age and sex;

Model 2: adjusted for Model 1 plus educational level, living alone, smoking status, alcohol consumption, total energy intake, physical activity level, medical history of hypertension, diabetes and heart diseases, and probable dementia.

**Table S7.** Sex-specific association between per SD increase in dietary pattern scores and incident sarcopenic obesity risk.

|  | **Men** | |  | **Women** | |
| --- | --- | --- | --- | --- | --- |
|  | **HR (95% CI)** | ***p*** |  | **HR (95% CI)** | ***p*** |
| No. of incident SO / Total | 131 / 1,686 |  |  | 135 / 1,712 |  |
| Incidence rate (per 1,000 person-years) | 21.06 |  |  | 21.45 |  |
| DQI-I | 0.857 (0.718–1.023) | 0.088 |  | 0.835 (0.703–0.992) | 0.041 |
| DII | 1.257 (1.032–1.531) | 0.023 |  | 1.063 (0.869–1.295) | 0.552 |
| MDS | 0.983 (0.823–1.173) | 0.850 |  | 0.954 (0.800–1.137) | 0.598 |
| DASH | 1.052 (0.881–1.256) | 0.573 |  | 0.936 (0.784–1.116) | 0.460 |
| MIND diet | 0.892 (0.745–1.069) | 0.219 |  | 0.809 (0.683–0.961) | 0.016 |
| Okinawa diet | 0.909 (0.759–1.089) | 0.304 |  | 0.815 (0.683–0.972) | 0.023 |
| *Hong Kong diet pattern* |  |  |  |  |  |
| Factor 1: vegetables-fruits | 0.996 (0.829–1.198) | 0.971 |  | 0.941 (0.785–1.126) | 0.507 |
| Factor 2: snacks-drinks-milk products | 0.945 (0.781–1.145) | 0.566 |  | 1.034 (0.854–1.252) | 0.734 |
| Factor 3: meat-fish | 0.910 (0.761–1.089) | 0.305 |  | 1.271 (1.081–1.495) | 0.004 |

Abbreviation: SD, standard deviation; HR, hazard ratio; CI, confidence interval; DQI-I, Diet Quality Index-International; DII, dietary inflammatory index; MDS, the Mediterranean diet score; DASH, the Dietary Approaches to Stop Hypertension; MIND, the Mediterranean-DASH Intervention for Neurodegenerative Delay Diet.

Adjusted for age, sex, educational level, living alone, smoking status, alcohol consumption, total energy intake, physical activity level, medical history of hypertension, diabetes and heart diseases, and probable dementia.

*p*-interactions between sex and dietary pattern scores in relation to incident SO risk were not statistically significant (all *p*-interactions >0.05).

**Table S8.** Sex-specific association between per SD increase in dietary pattern scores and incident sarcopenic obesity risk among individuals with baseline obesity.

|  | **From obesity to SO in men** | |  | **From obesity to SO in women** | |
| --- | --- | --- | --- | --- | --- |
|  | **HR (95% CI)** | ***p*** |  | **HR (95% CI)** | ***p*** |
| No. of incident SO / Total | 97/ 766 |  |  | 117 / 833 |  |
| Incidence rate (per 1,000 person-years) | 35.03 |  |  | 39.32 |  |
| DQI-I | 0.901 (0.733–1.109) | 0.325 |  | 0.840 (0.698–1.012) | 0.067 |
| DII | 1.257 (1.033–1.529) | 0.022 |  | 1.109 (0.914–1.347) | 0.291 |
| MDS | 1.000 (0.813–1.229) | 0.999 |  | 0.991 (0.827–1.187) | 0.921 |
| DASH | 1.047 (0.855–1.283) | 0.654 |  | 0.885 (0.732–1.071) | 0.210 |
| MIND diet | 0.882 (0.717–1.084) | 0.233 |  | 0.808 (0.672–0.971) | 0.023 |
| Okinawa diet | 0.938 (0.761–1.157) | 0.551 |  | 0.813 (0.677–0.978) | 0.028 |
| *Hong Kong diet pattern* |  |  |  |  |  |
| Factor 1: vegetables-fruits | 0.986 (0.795–1.224) | 0.901 |  | 0.940 (0.772–1.145) | 0.542 |
| Factor 2: snacks-drinks-milk products | 0.949 (0.758–1.191) | 0.656 |  | 0.982 (0.809–1.191) | 0.851 |
| Factor 3: meat-fish | 0.875 (0.713–1.072) | 0.197 |  | 1.224 (1.021–1.467) | 0.029 |

Abbreviation: SD, standard deviation; HR, hazard ratio; CI, confidence interval; DQI-I, Diet Quality Index-International; DII, dietary inflammatory index; MDS, the Mediterranean diet score; DASH, the Dietary Approaches to Stop Hypertension; MIND, the Mediterranean-DASH Intervention for Neurodegenerative Delay Diet.

Adjusted for age, sex, educational level, living alone, smoking status, alcohol consumption, physical activity level, total energy intake, medical history of hypertension, diabetes and heart diseases, and probable dementia in Cox regression model.

**Table S9.** Sensitivity analysis for association between per SD increase in dietary pattern scores and incident sarcopenic obesity risk over 2 years of follow-up.

|  | **Model 1** | |  | | **Model 2** | |
| --- | --- | --- | --- | --- | --- | --- |
|  | **HR (95% CI)** | ***p*** |  | | **HR (95% CI)** | ***p*** |
| No. of incident SO / Total | 177 / 3,260 | | |  | | |
| Incidence rate (per 1,000 person-years) | 26.89 | | |  | | |
| DQI-I | 0.858 (0.741–0.993) | 0.040 |  | | 0.891 (0.767–1.035) | 0.132 |
| DII | 1.189 (1.029–1.374) | 0.018 |  | | 1.115 (0.962–1.293) | 0.149 |
| MDS | 1.010 (0.872–1.169) | 0.892 |  | | 1.038 (0.893–1.206) | 0.628 |
| DASH | 1.009 (0.869–1.169) | 0.910 |  | | 1.013 (0.872–1.176) | 0.868 |
| MIND diet | 0.865 (0.749–0.999) | 0.048 |  | | 0.908 (0.784–1.051) | 0.194 |
| Okinawa diet | 0.924 (0.767–1.072) | 0.300 |  | | 0.959 (0.822–1.120) | 0.603 |
| *Hong Kong diet pattern* |  |  |  | |  |  |
| Factor 1: vegetables-fruits | 1.028 (0.890–1.187) | 0.703 |  | | 1.049 (0.907–1.214) | 0.521 |
| Factor 2: snacks-drinks-milk products | 0.966 (0.834–1.117) | 0.640 |  | | 1.009 (0.868–1.173) | 0.902 |
| Factor 3: meat-fish | 1.110 (0.963–1.279) | 0.149 |  | | 1.078 (0.933–1.244) | 0.307 |

Abbreviation: SD, standard deviation; HR, hazard ratio; CI, confidence interval; DQI-I, Diet Quality Index-International; DII, dietary inflammatory index; MDS, the Mediterranean diet score; DASH, the Dietary Approaches to Stop Hypertension; MIND, the Mediterranean-DASH Intervention for Neurodegenerative Delay Diet.

Model 1: adjusted for age and sex;

Model 2: adjusted for Model 1 plus educational level, living alone, smoking status, alcohol consumption, total energy intake, physical activity level, medical history of hypertension, diabetes and heart diseases, and probable dementia.

**Table S10.** Sensitivity analysis for association between per SD increase in dietary pattern scores and incident sarcopenic obesity risk among individuals with baseline obesity over 2 years of follow-up.

|  | **From obesity to SO** | |
| --- | --- | --- |
|  | **HR (95% CI)** | ***p*** |
| No. of incident SO / Total | 155 / 1,547 |  |
| Incidence rate (per 1,000 person-years) | 49.66 |  |
| DQI-I | 0.841 (0.712–0.994) | 0.042 |
| DII | 1.251 (1.042–1.501) | 0.016 |
| MDS | 0.983 (0.829–1.166) | 0.844 |
| DASH | 0.932 (0.794–1.094) | 0.392 |
| MIND diet | 0.859 (0.732–1.008) | 0.062 |
| Okinawa diet | 0.909 (0.773–1.070) | 0.253 |
| *Hong Kong diet pattern* |  |  |
| Factor 1: vegetables-fruits | 0.992 (0.845–1.166) | 0.928 |
| Factor 2: snacks-drinks-milk products | 1.017 (0.865–1.197) | 0.834 |
| Factor 3: meat-fish | 1.077 (0.921–1.258) | 0.352 |

Abbreviation: SD, standard deviation; HR, hazard ratio; CI, confidence interval; DQI-I, Diet Quality Index-International; DII, dietary inflammatory index; MDS, the Mediterranean diet score; DASH, the Dietary Approaches to Stop Hypertension; MIND, the Mediterranean-DASH Intervention for Neurodegenerative Delay Diet.

Adjusted for age, sex, educational level, living alone, smoking status, alcohol consumption, physical activity level, total energy intake, medical history of hypertension, diabetes and heart diseases, and probable dementia in Cox regression model.

**Table S11.** Association between per SD increase in dietary pattern scores and incident sarcopenic obesity risk before and after IPCW.

|  | **Main analysis (unweighted)** | |  | **IPCW analysis**  **(weighted)** | |
| --- | --- | --- | --- | --- | --- |
|  | **HR (95% CI)** | ***p*** |  | **HR (95% CI)** | ***p*** |
| DQI-I | 0.858 (0.756–0.973) | 0.017 |  | 0.853 (0.749–0.971) | 0.017 |
| DII | 1.165 (1.005–1.349) | 0.043 |  | 1.174 (1.011–1.362) | 0.035 |
| MDS | 0.963 (0.853–1.088) | 0.547 |  | 0.967 (0.856–1.093) | 0.597 |
| DASH | 1.015 (0.892–1.155) | 0.817 |  | 1.001 (0.879–1.141) | 0.979 |
| MIND diet | 0.859 (0.762–0.969) | 0.014 |  | 0.853 (0.755–0.964) | 0.011 |
| Okinawa diet | 0.868 (0.767–0.982) | 0.025 |  | 0.863 (0.761–0.978) | 0.021 |
| *Hong Kong diet pattern* |  |  |  |  |  |
| Factor 1: vegetables-fruits | 0.971 (0.849–1.111) | 0.672 |  | 0.973 (0.850–1.114) | 0.694 |
| Factor 2: snacks-drinks-milk products | 0.996 (0.868–1.141) | 0.949 |  | 0.956 (0.858–1.133) | 0.839 |
| Factor 3: meat-fish | 1.067 (0.942, 1.208) | 0.306 |  | 1.074 (0.948–1.218) | 0.262 |

Abbreviation: SD, standard deviation; IPCW, Inverse Probability of Censoring Weighting; HR, hazard ratio; CI, confidence interval; DQI-I, Diet Quality Index-International; DII, dietary inflammatory index; MDS, the Mediterranean diet score; DASH, the Dietary Approaches to Stop Hypertension; MIND, the Mediterranean-DASH Intervention for Neurodegenerative Delay Diet.

Adjusted for age, sex, educational level, living alone, smoking status, alcohol consumption, total energy intake, physical activity level, medical history of hypertension, diabetes and heart diseases, and probable dementia.

Main analysis (unweighted): Cox proportional hazards regression models were performed among the 3,398 participants included in the final analysis.

IPCW analysis (weighted): IPCW was applied to account for potential selection bias due to missing data or loss to follow-up. The stabilized weights were calculated using a multivariable logistic regression model predicting the probability of being included in the final analytical sample, based on the entire baseline cohort.

**Table S12.** Association between per SD increase in dietary pattern scores and incident sarcopenic obesity risk among individuals with baseline obesity before and after IPCW.

|  | **From obesity to SO** | | | | |
| --- | --- | --- | --- | --- | --- |
|  | **Main analysis (unweighted)** | |  | **IPCW analysis**  **(weighted)** | |
|  | **HR (95% CI)** | ***p*** |  | **HR (95% CI)** | ***p*** |
| DQI-I | 0.866 (0.752–0.996) | 0.044 |  | 0.860 (0.743–0.996) | 0.045 |
| DII | 1.223 (1.046–1.429) | 0.011 |  | 1.245 (1.052–1.473) | 0.011 |
| MDS | 0.989 (0.858–1.140) | 0.881 |  | 0.993 (0.863–1.144) | 0.928 |
| DASH | 0.977 (0.851–1.121) | 0.742 |  | 0.976 (0.843–1.131) | 0.751 |
| MIND diet | 0.835 (0.728–0.959) | 0.011 |  | 0.839 (0.731–0.961) | 0.012 |
| Okinawa diet | 0.858 (0.747–0.984) | 0.029 |  | 0.854 (0.744–0.979) | 0.024 |
| *Hong Kong diet pattern* |  |  |  |  |  |
| Factor 1: vegetables-fruits | 0.968 (0.837–1.118) | 0.658 |  | 0.966 (0.825–1.131) | 0.670 |
| Factor 2: snacks-drinks-milk products | 0.964 (0.829–1.121) | 0.634 |  | 0.962 (0.832–1.112) | 0.599 |
| Factor 3: meat-fish | 1.048 (0.916–1.200) | 0.491 |  | 1.041 (0.904–1.199) | 0.578 |

Abbreviation: SD, standard deviation; IPCW, Inverse Probability of Censoring Weighting; HR, hazard ratio; CI, confidence interval; DQI-I, Diet Quality Index-International; DII, dietary inflammatory index; MDS, the Mediterranean diet score; DASH, the Dietary Approaches to Stop Hypertension; MIND, the Mediterranean-DASH Intervention for Neurodegenerative Delay Diet.

Adjusted for age, sex, educational level, living alone, smoking status, alcohol consumption, total energy intake, physical activity level, medical history of hypertension, diabetes and heart diseases, and probable dementia.

Main analysis (unweighted): Standard Cox proportional hazards regression models were performed among the 1,599 participants with baseline obesity included in the final analysis.

IPCW analysis (weighted): IPCW was applied to account for potential selection bias due to missing data or loss to follow-up. The stabilized weights were calculated using a multivariable logistic regression model predicting the probability of being included in the final analytical sample, based on the entire baseline cohort.

**Table S13.** Sensitivity analysis for association between per SD increase in dietary pattern scores and incident sarcopenic obesity risk using Fine-Gray competing risk model.

|  | **Model 1** | |  | **Model 2** | |
| --- | --- | --- | --- | --- | --- |
|  | **SHR (95% CI)** | ***p*** |  | **SHR (95% CI)** | ***p*** |
| No. of incident SO / Total | 266 / 3,398 |  |  |  |  |
| Incidence Rate (per 1,000 person-years) | 21.26 |  |  |  |  |
| No. of competing deaths | 95 |  |  |  |  |
| DQI-I | 0.837 (0.742–0.945) | 0.004 |  | 0.858 (0.756–0.973) | 0.017 |
| DII | 1.183 (1.038–1.348) | 0.012 |  | 1.165 (1.005–1.349) | 0.043 |
| MDS | 0.935 (0.830–1.052) | 0.263 |  | 0.963 (0.853–1.088) | 0.547 |
| DASH | 1.005 (0.884–1.141) | 0.994 |  | 1.015 (0.892–1.155) | 0.817 |
| MIND diet | 0.836 (0.742–0.944) | 0.003 |  | 0.859 (0.762–0.969) | 0.014 |
| Okinawa diet | 0.845 (0.750–0.952) | 0.006 |  | 0.868 (0.767–0.982) | 0.025 |
| *Hong Kong diet pattern* |  |  |  |  |  |
| Factor 1: vegetables-fruits | 0.953 (0.833–1.089) | 0.482 |  | 0.971 (0.849–1.111) | 0.672 |
| Factor 2: snacks-drinks-milk products | 0.948 (0.831–1.081) | 0.424 |  | 0.996 (0.868–1.141) | 0.949 |
| Factor 3: meat-fish | 1.084 (0.963–1.221) | 0.182 |  | 1.067 (0.942, 1.208) | 0.306 |

Abbreviation: SD, standard deviation; SHR, subdistribution hazard ratio; CI, confidence interval; DQI-I, Diet Quality Index-International; DII, dietary inflammatory index; MDS, the Mediterranean diet score; DASH, the Dietary Approaches to Stop Hypertension; MIND, the Mediterranean-DASH Intervention for Neurodegenerative Delay Diet.

Model 1: adjusted for age and sex;

Model 2: adjusted for Model 1 plus educational level, living alone, smoking status, alcohol consumption, total energy intake, physical activity level, medical history of hypertension, diabetes and heart diseases, and probable dementia.

**Table S14.** Sensitivity analysis for association between per SD increase in dietary pattern scores and incident sarcopenic obesity risk among individuals with baseline obesity using Fine-Gray competing risk model.

|  | **From obesity to SO** | |
| --- | --- | --- |
|  | **SHR (95% CI)** | ***p*** |
| No. of incident SO / Total | 214 / 1,599 |  |
| Incidence Rate (per 1,000 person-years) | 37.28 |  |
| No. of competing deaths | 35 |  |
| DQI-I | 0.866 (0.751–0.999) | 0.048 |
| DII | 1.240 (1.049–1.465) | 0.011 |
| MDS | 0.977 (0.850–1.124) | 0.750 |
| DASH | 0.992 (0.857–1.148) | 0.915 |
| MIND diet | 0.843 (0.737–0.963) | 0.012 |
| Okinawa diet | 0.853 (0.745–0.977) | 0.022 |
| *Hong Kong diet pattern* |  |  |
| Factor 1: vegetables-fruits | 0.961 (0.822–1.124) | 0.622 |
| Factor 2: snacks-drinks-milk products | 0.967 (0.839–1.114) | 0.640 |
| Factor 3: meat-fish | 1.045 (0.908–1.203) | 0.536 |

Abbreviation: SD, standard deviation; SHR, subdistribution hazard ratio; CI, confidence interval; DQI-I, Diet Quality Index-International; DII, dietary inflammatory index; MDS, the Mediterranean diet score; DASH, the Dietary Approaches to Stop Hypertension; MIND, the Mediterranean-DASH Intervention for Neurodegenerative Delay Diet.

Adjusted for age, sex, educational level, living alone, smoking status, alcohol consumption, physical activity level, total energy intake, medical history of hypertension, diabetes and heart diseases, and probable dementia in Cox regression model.

**Table S15**. Different criteria for sarcopenia and obesity.

|  | **Sarcopenia** | **Obesity** |
| --- | --- | --- |
| **1.** | **AWGS 2025 criteria (low muscle mass with low muscle strength or/and low physical performance):**   (1) Low muscle strength:   Handgrip strength <28 kg for men and <18 kg for women   (2) Low physical performance:   Gait speed: <1.0 m/s; or  five-time chair stand test: ≥12 s; or  SPPB: ≤9   (3) Low muscle mass:   ASMI <7.0 kg/m^2^ for men and <5.4 kg/m^2^ for women (DXA) | **AACE/ACE and WHO criteria:**  BFP ≥25% for men and ≥35% for women |
| **2.** | **AWGS 2019 criteria (low muscle mass and low muscle strength):**   (1) Low muscle strength:   Handgrip strength <28 kg for men and <18 kg for women   (2) Low muscle mass:   ASMI <7.0 kg/m^2^ for men and <5.4 kg/m^2^ for women; or  ASM/BMI <0.83 for men and <0.57 for women (DXA) | BFP ≥ 60^th^ percentile in the study population (≥25.9% for men, ≥36.2% for women) |
| **3.** |  | **WHO criteria for Asian populations:**  BMI≥27.5 kg/m^2^, or  WC ≥90 cm for men, ≥80 cm for women |

Abbreviation: AWGS, Asian Working Group for Sarcopenia; AACE, American Association of Clinical Endocrinology; ACE, American College of Endocrinology; WHO, world health organization; BFP, body fat percentage; SPPB, the Short Physical Performance Battery; DXA, dual-energy X-ray absorptiometry; BMI, body mass index; BPF, body fat percentage; ASMI, appendicular skeletal muscle mass index; WC, waist circumference.

**Figure S2**. Venn diagram showing the overlap of incident SO cases defined by AWGS 2019 or AWGS 2025 criteria.

Overlap of incident SO cases identified by the AWGS 2019 or updated AWGS 2025 criteria with ≥25% for men and ≥35% for women. The blue circle represents cases identified by AWGS 2019 (n=233), while the orange circle represents cases identified by AWGS 2025 (n=266). The intersection indicates individuals meeting both criteria (n=81).

**Table S16.** Sensitivity analysis for association between per SD increase in dietary pattern scores and incident sarcopenic obesity risk based on different categories of sarcopenia and obesity.

|  | **Model 1** | |  | **Model 2** | |
| --- | --- | --- | --- | --- | --- |
|  | **HR (95% CI)** | ***p*** |  | **HR (95% CI)** | ***p*** |
| **AWGS 2025 + BFP ≥60^th^ percentile (≥25.9% for men, ≥36.2% for women)** | | | | | |
| Number of incident cases/ N | 234 / 3,419 |  |  |  |  |
| Incidence Rate (per 1,000 person-years) | 18.55 |  |  |  |  |
| DQI-I | 0.848 (0.748–0.962) | 0.010 |  | 0.869 (0.761–0.991) | 0.037 |
| DII | 1.163 (1.024–1.321) | 0.020 |  | 1.127 (0.989–1.283) | 0.072 |
| MDS | 0.925 (0.815–1.051) | 0.232 |  | 0.958 (0.839–1.094) | 0.529 |
| DASH | 0.949 (0.832–1.083) | 0.443 |  | 0.962 (0.842–1.098) | 0.566 |
| MIND diet | 0.826 (0.726–0.938) | 0.003 |  | 0.849 (0.745–0.968) | 0.015 |
| Okinawa diet | 0.903 (0.794–1.026) | 0.118 |  | 0.926 (0.811–1.057) | 0.257 |
| *Hong Kong diet pattern* |  |  |  |  |  |
| Factor 1: vegetables-fruits | 0.963 (0.844–1.098) | 0.573 |  | 0.986 (0.863–1.127) | 0.842 |
| Factor 2: snacks-drinks-milk products | 0.979 (0.858–1.118) | 0.762 |  | 1.013 (0.881–1.166) | 0.851 |
| Factor 3: meat-fish | 1.075 (0.948–1.219) | 0.258 |  | 1.059 (0.930–1.205) | 0.387 |
| **AWGS 2025 + BMI ≥27.5 kg/m², or WC ≥90 cm for men, ≥80 cm for women** | | | | | |
| Number of incident cases/ N | 222 / 3,441 |  |  |  |  |
| Incidence Rate (per 1,000 person-years) | 17.44 |  |  |  |  |
| DQI-I | 0.823 (0.725–0.934) | 0.003 |  | 0.862 (0.753–0.986) | 0.030 |
| DII | 1.179 (1.034–1.344) | 0.014 |  | 1.066 (0.933–1.219) | 0.346 |
| MDS | 0.971 (0.851–1.107) | 0.660 |  | 1.018 (0.888–1.167) | 0.794 |
| DASH | 0.923 (0.805–1.058) | 0.248 |  | 0.939 (0.818–1.078) | 0.373 |
| MIND diet | 0.803 (0.704–0.915) | 0.001 |  | 0.840 (0.735–0.961) | 0.011 |
| Okinawa diet | 0.855 (0.749–0.976) | 0.020 |  | 0.897 (0.783–1.029) | 0.120 |
| *Hong Kong diet pattern* |  |  |  |  |  |
| Factor 1: vegetables-fruits | 0.993 (0.958–1.029) | 0.715 |  | 1.001 (0.966–1.037) | 0.960 |
| Factor 2: snacks-drinks-milk products | 0.908 (0.787–1.049) | 0.190 |  | 0.992 (0.853–1.154) | 0.917 |
| Factor 3: meat-fish | 1.114 (0.981–1.265) | 0.096 |  | 1.094 (0.959–1.246) | 0.178 |
| **AWGS 2019 + BFP ≥25% for men, ≥35% for women** | | | | | |
| Number of incident cases/ N | 233 / 3,212 |  |  |  |  |
| Incidence Rate (per 1,000 person-years) | 19.69 |  |  |  |  |
| DQI-I | 0.896 (0.789–1.016) | 0.088 |  | 0.903 (0.790–1.031) | 0.132 |
| DII | 1.127 (0.989–1.284) | 0.072 |  | 1.096 (0.960–1.252) | 0.173 |
| MDS | 0.887 (0.782–1.009) | 0.066 |  | 0.916 (0.803–1.046) | 0.195 |
| DASH | 1.058 (0.929–1.204) | 0.391 |  | 1.067 (0.937–1.216) | 0.325 |
| MIND diet | 0.941 (0.827–1.070) | 0.352 |  | 0.954 (0.836–1.090) | 0.494 |
| Okinawa diet | 0.923 (0.812–1.049) | 0.219 |  | 0.936 (0.819–1.069) | 0.328 |
| *Hong Kong diet pattern* |  |  |  |  |  |
| Factor 1: vegetables-fruits | 0.971 (0.851–1.109) | 0.665 |  | 0.982 (0.857–1.124) | 0.790 |
| Factor 2: snacks-drinks-milk products | 1.035 (0.909–1.178) | 0.602 |  | 1.040 (0.905–1.195) | 0.580 |
| Factor 3: meat-fish | 1.038 (0.915–1.178) | 0.558 |  | 1.042 (0.914–1.187) | 0.541 |

Abbreviation: SD, standard deviation; HR, hazard ratio; CI, confidence interval; AWGS, Asian Working Group for Sarcopenia; BFP, body fat percentage; BMI, body mass index; BPF, body fat percentage; ASMI, appendicular skeletal muscle mass index; WC, waist circumference; DQI-I, Diet Quality Index-International; DII, dietary inflammatory index; MDS, the Mediterranean diet score; DASH, the Dietary Approaches to Stop Hypertension; MIND, the Mediterranean-DASH Intervention for Neurodegenerative Delay Diet.

Adjusted for age, sex, educational level, living alone, smoking status, alcohol consumption, physical activity level, total energy intake, medical history of hypertension, diabetes and heart diseases, and probable dementia in Cox regression model.

**Table S17.** Sensitivity analysis for association between per SD increase in dietary pattern scores and incident sarcopenic obesity risk among individuals with baseline obesity based on different categories of sarcopenia and obesity.

|  | **From obesity to SO** | |
| --- | --- | --- |
|  | **HR (95% CI)** | ***p*** |
| **AWGS 2025 + BFP ≥60th percentile (≥25.9% for men, ≥36.2% for women)** | | |
| Incident SO / Total | 186 / 1,295 |  |
| Incidence Rate (per 1,000 person-years) | 40.14 |  |
| DQI-I | 0.883 (0.760–0.997) | 0.046 |
| DII | 1.156 (0.995–1.342) | 0.057 |
| MDS | 0.998 (0.856–1.163) | 0.976 |
| DASH | 0.949 (0.814–1.106) | 0.504 |
| MIND diet | 0.835 (0.718–0.969) | 0.018 |
| Okinawa diet | 0.898 (0.777–1.038) | 0.145 |
| *Hong Kong diet pattern* |  |  |
| Factor 1: vegetables-fruits | 1.019 (0.875–1.187) | 0.806 |
| Factor 2: snacks-drinks-milk products | 0.983 (0.844–1.146) | 0.832 |
| Factor 3: meat-fish | 1.055 (0.912–1.221) | 0.471 |
| **AWGS 2025 + BMI ≥27.5 kg/m², or WC ≥90 cm for men, ≥80 cm for women** | | |
| Incident SO / Total | 165 / 1,905 |  |
| Incidence Rate (per 1,000 person-years) | 23.61 |  |
| DQI-I | 0.803 (0.687–0.938) | 0.006 |
| DII | 1.136 (0.971–1.328) | 0.111 |
| MDS | 1.036 (0.883–1.217) | 0.662 |
| DASH | 0.959 (0.815–1.130) | 0.624 |
| MIND diet | 0.768 (0.659–0.896) | 0.001 |
| Okinawa diet | 0.818 (0.698–0.959) | 0.014 |
| *Hong Kong diet pattern* |  |  |
| Factor 1: vegetables-fruits | 1.001 (0.959–1.044) | 0.971 |
| Factor 2: snacks-drinks-milk products | 0.887 (0.733–1.074) | 0.220 |
| Factor 3: meat-fish | 1.069 (0.915–1.249) | 0.401 |
| **AWGS 2019 + BFP ≥25% for men, ≥35% for women** | | |
| Incident SO / Total | 153 / 1,430 |  |
| Incidence Rate (per 1,000 person-years) | 29.41 |  |
| DQI-I | 0.948 (0.802–1.121) | 0.534 |
| DII | 1.085 (0.920–1.279) | 0.331 |
| MDS | 0.892 (0.751–1.059) | 0.193 |
| DASH | 1.155 (0.987–1.351) | 0.073 |
| MIND diet | 0.937 (0.794–1.105) | 0.439 |
| Okinawa diet | 0.982 (0.835–1.154) | 0.823 |
| *Hong Kong diet pattern* |  |  |
| Factor 1: vegetables-fruits | 1.053 (0.901–1.232) | 0.513 |
| Factor 2: snacks-drinks-milk products | 0.955 (0.799–1.142) | 0.615 |
| Factor 3: meat-fish | 1.036 (0.881–1.218) | 0.669 |

Abbreviation: SD, standard deviation; HR, hazard ratio; CI, confidence interval; AWGS, Asian Working Group for Sarcopenia; BFP, body fat percentage; BMI, body mass index; BPF, body fat percentage; ASMI, appendicular skeletal muscle mass index; WC, waist circumference; DQI-I, Diet Quality Index-International; DII, dietary inflammatory index; MDS, the Mediterranean diet score; DASH, the Dietary Approaches to Stop Hypertension; MIND, the Mediterranean-DASH Intervention for Neurodegenerative Delay Diet.

Adjusted for age, sex, educational level, living alone, smoking status, alcohol consumption, physical activity level, total energy intake, medical history of hypertension, diabetes and heart diseases, and probable dementia in Cox regression model.

**Table S18.** Sensitivity analysis for association between per SD increase in dietary pattern scores and incident sarcopenic obesity risk.

|  | **Model 3** | |  | **Model 4** | |
| --- | --- | --- | --- | --- | --- |
|  | **HR (95% CI)** | ***p*** |  | **HR (95% CI)** | ***p*** |
| DQI-I | 0.882 (0.762, 1.022) | 0.094 |  | 0.831 (0.724, 0.951) | 0.007 |
| DII | 1.205 (1.022, 1.421) | 0.026 |  | 1.138 (0.972, 1.333) | 0.108 |
| MDS | 0.979 (0.843, 1.135) | 0.777 |  | 0.925 (0.784, 1.090) | 0.353 |
| DASH | 1.026 (0.884, 1.191) | 0.730 |  | 0.967 (0.842, 1.112) | 0.639 |
| MIND diet | 0.853 (0.737, 0.987) | 0.033 |  | 0.834 (0.737, 0.957) | 0.010 |
| Okinawa diet | 0.839 (0.719, 0.978) | 0.025 |  | 0.847 (0.735, 0.975) | 0.021 |
| *Hong Kong diet pattern* |  |  |  |  |  |
| Factor 1: vegetables-fruits | 0.972 (0.833, 1.135) | 0.723 |  | 0.954 (0.827, 1.101) | 0.520 |
| Factor 2: snacks-drinks-milk products | 1.026 (0.869, 1.210) | 0.763 |  | 1.065 (0.919, 1.232) | 0.402 |
| Factor 3: meat-fish | 1.106 (0.957, 1.277) | 0.169 |  | 1.163 (1.020, 1.326) | 0.024 |

Abbreviation: SD, standard deviation; HR, hazard ratio; CI, confidence interval; DQI-I, Diet Quality Index-International; DII, dietary inflammatory index; MDS, the Mediterranean diet score; DASH, the Dietary Approaches to Stop Hypertension; MIND, the Mediterranean-DASH Intervention for Neurodegenerative Delay Diet.

Model 2: adjusted for age, sex, educational level, living alone, smoking status, alcohol consumption, physical activity level, total energy intake, medical history of hypertension, diabetes and heart diseases, and probable dementia in Cox regression model.

Model 3: adjusted for Model 2 plus log-transformed serum hs-CRP levels.

Model 4: adjusted for Model 2 plus eGFR.

**Table S19.** Sensitivity analysis for the association between per SD increase in dietary pattern scores and incident sarcopenic obesity risk among individuals with baseline obesity.

|  | **From obesity to SO** | | | | |
| --- | --- | --- | --- | --- | --- |
|  | **Model 3** | |  | **Model 4** | |
|  | **HR (95% CI)** | ***p*** |  | **HR (95% CI)** | ***p*** |
| DQI-I | 0.917 (0.776, 1.085) | 0.313 |  | 0.843 (0.719, 0.987) | 0.033 |
| DII | 1.284 (1.070, 1.542) | 0.007 |  | 1.232 (1.031, 1.471) | 0.021 |
| MDS | 0.995 (0.841, 1.178) | 0.959 |  | 0.923 (0.788, 1.080) | 0.318 |
| DASH | 1.050 (0.891, 1.237) | 0.560 |  | 0.921 (0.787, 1.078) | 0.305 |
| MIND diet | 0.843 (0.716, 0.993) | 0.041 |  | 0.804 (0.689, 0.939) | 0.006 |
| Okinawa diet | 0.841 (0.715, 0.990) | 0.038 |  | 0.840 (0.720, 0.980) | 0.027 |
| *Hong Kong diet pattern* |  |  |  |  |  |
| Factor 1: vegetables-fruits | 0.943 (0.792, 1.123) | 0.514 |  | 0.930 (0.787, 1.099) | 0.395 |
| Factor 2: snacks-drinks-milk products | 0.943 (0.792, 1.123) | 0.514 |  | 0.930 (0.787, 1.099) | 0.395 |
| Factor 3: meat-fish | 1.036 (0.881, 1.216) | 0.668 |  | 1.131 (0.975, 1.311) | 0.102 |

Abbreviation: SD, standard deviation; HR, hazard ratio; CI, confidence interval; DQI-I, Diet Quality Index-International; DII, dietary inflammatory index; MDS, the Mediterranean diet score; DASH, the Dietary Approaches to Stop Hypertension; MIND, the Mediterranean-DASH Intervention for Neurodegenerative Delay Diet.

Model 2: adjusted for age, sex, educational level, living alone, smoking status, alcohol consumption, physical activity level, total energy intake, medical history of hypertension, diabetes and heart diseases, and probable dementia in Cox regression model.

Model 3: adjusted for Model 2 plus log-transformed serum hs-CRP levels.

Model 4: adjusted for Model 2 plus eGFR.

**Figure S3.** Subgroup analysis for association between per SD increase in dietary pattern scores and incident sarcopenic obesity risk based on the median of serum hs-CRP or eGFR.

Adjusted for age, sex, educational level, living alone, smoking status, alcohol consumption, physical activity level, total energy intake, medical history of hypertension, diabetes and heart diseases, and probable dementia in Cox regression model. * The interaction between DII and eGFR in relation to incident SO was significant (*p*-interaction <0.001); but no significant interactions for other dietary patterns with serum hs-CRP or eGFR were observed.

Abbreviation: hs-CRP, high-sensitivity C-reactive protein; eGFR, estimated glomerular filtration rate; SD, standard deviation; SO, sarcopenic obesity; HR, hazard ratio; CI, confidence interval; DQI-I, Diet Quality Index-International; DII, dietary inflammatory index; MDS, the Mediterranean diet score; DASH, the Dietary Approaches to Stop Hypertension; MIND, the Mediterranean-DASH Intervention for Neurodegenerative Delay Diet.

**Table S20.** Associations between each dietary pattern score with log-transformed serum hs-CRP levels and eGFR in multivariable adjusted linear regression model.

|  | **Serum hs-CRP** | | | |  | **eGFR** | | | |
| --- | --- | --- | --- | --- | --- | --- | --- | --- | --- |
|  | **Age- and sex-adjusted** | | **Multivariate adjusted** | |  | **Age- and sex-adjusted** | | **Multivariate adjusted** | |
|  | **β (95% CI)** | ***p*** | **β (95% CI)** | ***p*** |  | **β (95% CI)** | ***p*** | **β (95% CI)** | ***p*** |
| DQI-I | −0.077 (−0.122, −0.031) | 0.001 | −0.045 (−0.092, 0.002) | 0.059 |  | 1.121 (0.651, 1.590) | <0.001 | 1.111 (0.628, 1.593) | <0.001 |
| DII | 0.068 (0.023, 0.114) | 0.003 | 0.060 (0.014, 0.106) | 0.010 |  | −1.005 (−1.476, −0.534) | <0.001 | −1.052 (−1.598, −0.507) | <0.001 |
| MDS | −0.029 (−0.076, 0.017) | 0.220 | −0.011 (−0.059, 0.036) | 0.641 |  | 0.966 (0.504, 1.428) | <0.001 | 0.876 (0.404, 1.349) | <0.001 |
| DASH | −0.054 (−0.099, −0.008) | 0.021 | −0.049 (−0.094, −0.003) | 0.037 |  | 0.875 (0.405, 1.343) | <0.001 | 0.917 (0.452, 1.382) | <0.001 |
| MIND diet | −0.057 (−0.103, −0.011) | 0.014 | −0.049 (−0.094, −0.003) | 0.037 |  | 0.561 (0.090, 1.033) | 0.020 | 0.554 (0.075, 1.032) | 0.023 |
| Okinawa diet | −0.042 (−0.088, 0.004) | 0.071 | −0.030 (−0.077, 0.017) | 0.205 |  | 0.261 (−0.205, 0.728) | 0.272 | 0.162 (−0.312, 0.635) | 0.504 |
| ***Hong Kong diet pattern*** |  |  |  |  |  |  |  |  |  |
| Factor 1: vegetables-fruits | −0.029 (−0.075, 0.017) | 0.212 | −0.012 (−0.059, 0.034) | 0.599 |  | 0.930 (0.460, 1.400) | <0.001 | 1.025 (0.555, 1.495) | <0.001 |
| Factor 2: snacks-drinks-milk products | −0.066 (−0.113, −0.019) | 0.005 | −0.058 (−0.108, −0.009) | 0.020 |  | 0.405 (−0.065, 0.876) | 0.091 | 0.306 (−0.189, 0.802) | 0.226 |
| Factor 3: meat-fish | 0.063 (0.017, 0.109) | 0.007 | 0.061 (0.014, 0.107) | 0.010 |  | −0.420 (−0.885, 0.044) | 0.076 | −0.453 (−0.918, 0.012) | 0.056 |

Abbreviation: hs-CRP, high-sensitivity C-reactive protein; eGFR, estimated glomerular filtration rate; DQI-I, Diet Quality Index-International; DII, dietary inflammatory index; MDS, the Mediterranean diet score; DASH, the Dietary Approaches to Stop Hypertension; MIND, the Mediterranean-DASH Intervention for Neurodegenerative Delay Diet.

The linear regression model was adjusted for age, sex, educational level, living alone, smoking status, alcohol consumption, total energy intake, physical activity level, medical history of hypertension, diabetes and heart diseases, and probable dementia. The coefficients with 95% CI were estimated.
